# Brain Derived Neurotrophic Factor Contributes to the Cardiogenic Potential of Adult Resident Progenitor Cells in Failing Murine Heart

**DOI:** 10.1371/journal.pone.0120360

**Published:** 2015-03-23

**Authors:** Rasmita Samal, Sabine Ameling, Vishnu Dhople, Praveen Kumar Sappa, Kristin Wenzel, Uwe Völker, Stephan B. Felix, Elke Hammer, Stephanie Könemann

**Affiliations:** 1 Department of Internal Medicine B, University Medicine Greifswald, Greifswald, Germany; 2 Interfaculty Institute for Genetics and Functional Genomics, University Medicine Greifswald, Greifswald, Germany; 3 DZHK (German Center for Cardiovascular Research) partner site, Greifswald, Germany; Georgia Regents University, UNITED STATES

## Abstract

**Aims:**

Resident cardiac progenitor cells show homing properties when injected into the injured but not to the healthy myocardium. The molecular background behind this difference in behavior needs to be studied to elucidate how adult progenitor cells can restore cardiac function of the damaged myocardium. Since the brain derived neurotrophic factor (BDNF) moderates cardioprotection in injured hearts, we focused on delineating its regulatory role in the damaged myocardium.

**Methods and Results:**

Comparative gene expression profiling of freshly isolated undifferentiated Sca-1 progenitor cells derived either from heart failure transgenic αMHC-CyclinT1/Gαq overexpressing mice or wildtype littermates revealed transcriptional variations. Bdnf expression was up regulated 5-fold during heart failure which was verified by qRT-PCR and confirmed at protein level. The migratory capacity of Sca-1 cells from transgenic hearts was improved by 15% in the presence of 25ng/ml BDNF. Furthermore, BDNF-mediated effects on Sca-1 cells were studied via pulsed Stable Isotope Labeling of Amino acids in Cell Culture (pSILAC) proteomics approach. After BDNF treatment significant differences between newly synthesized proteins in Sca-1 cells from control and transgenic hearts were observed for CDK1, SRRT, HDGF, and MAP2K3 which are known to regulate cell cycle, survival and differentiation. Moreover BDNF repressed the proliferation of Sca-1 cells from transgenic hearts.

**Conclusion:**

Comparative profiling of resident Sca-1 cells revealed elevated BDNF levels in the failing heart. Exogenous BDNF (i) stimulated migration, which might improve the homing ability of Sca-1 cells derived from the failing heart and (ii) repressed the cell cycle progression suggesting its potency to ameliorate heart failure.

## Introduction

Despite various attempts to develop therapeutics for cardiac disorders, the prevalence of heart failure was not considerably reduced. Moreover, the number of patients with heart failure is still growing due to demographic changes and higher survival rate after acute myocardial infarction. Although enormous progress has been made in the field of cardiovascular research, heart transplantation remains the solitary cure for end-stage heart failure till today. However, lack of donor hearts, tissue rejection and the high costs of treatment are major limitations in meeting the increasing demand of patients and foster the search for new treatment options. Over the last decade cell-based therapies emerged as potential alternatives in this regard. Accumulating evidence shows that a subset of undifferentiated progenitor cell populations resides in the adult heart, which is capable of promoting regeneration of the damaged myocardium [1–3] and thus offers new options towards endogenous cardiac repair mechanisms.

Pioneering work by the group of M. Schneider has described cardiac primitive cells that expressed stem cell antigen-1 (Sca-1) on their surface comprising 14–17% of the non-myocyte adult cardiac cell population [4]. Although the human homologue of Sca-1 is still unknown, a previously reported study has shown that human hematopoietic stem cells transduced with mouse Sca-1 showed similar myeloid colony forming ability as their mouse counterparts suggesting the existence of functional orthologues of Sca-1 in humans [5]. Sca-1 was reported to promote cardiac stem cell proliferation and survival facilitating early engraftment and late cardiovascular differentiation [6]. In our previous study, the molecular identity of undifferentiated Sca-1 cells was reported in more detail [7]. Adult cardiac progenitor cells remain quiescent under physiological conditions unless challenged by myocardial insult. Although efforts have been made to characterize adult progenitor cells, the molecular alterations that occur during heart failure and then in turn alter the functional properties of adult progenitor cells are largely unknown.

Microarray-based global transcriptome analysis can provide deeper insight into the regulatory mechanisms of diseases [8]. Most recently, the molecular relationship among various progenitor cells (ckit+, Sca-1+, side population) derived from adult myocardium has been characterized with the aid of microarrays [9]. However, little is known about the transcriptional variations in adult resident Sca-1 cells derived from failing hearts in comparison to cells from healthy organs. Hence, the identification of regulatory factors that influence the progression of diseases would be a first step towards the exploration of their therapeutic potential under pathophysiological conditions.

In the present study, we investigated the molecular differences in adult Sca-1 cells under heart failure condition compared to normal cells to gain better insight into their cardiogenic potential in a pathological milieu. In Sca-1cells derived from failing heart, the expression of BDNF was significantly enhanced when compared to cells from healthy hearts. Although neuroprotective function of BDNF in promoting growth, survival and regeneration of nervous system has been extensively reported [10,11], it is evident that BDNF is also essential for cardiovascular development [12,13]. BDNF is known to play a protective role in heart by inducing angiogenesis and upregulation of prosurvival factors[14] and also promote neovascularization of ischemic tissue by recruitment of endothelial cells[15]. However the underlying mechanism of BDNF action in cardiac dysfunction is not clear. Since BDNF-treatment might constitute a therapeutic option for treatment of end stage heart failure, we tested the impact of BDNF on cardiac homeostasis by a pulsed SILAC approach to quantitatively monitor proteins synthesized after treatment [16,17]. Hence, our results show that BDNF-mediated effect is not only limited to neural or endothelial cells but may also have an impact on cardiac progenitor cells and might contribute to cardiac repair.

## Methods

### Ethics statement

All animal experiments were performed in compliance with the Guide for the Care and Use of Laboratory Animals published by the U.S.NIH (NIH Publication no. 85–23, revised 1985) and the protocols were approved by the local animal care committee (Landesamt für Landwirtschaft, Lebensmittelsicherheit und Fischerei Mecklenburg-Vorpommern; LALLF MV).

According to the Declaration of Helsinki, HUVECs were isolated from the umbilical cords of patients from whom a written informed consent was received and the protocols were approved by the Ethics Committee of the University of Greifswald, Germany.

### Experimental animals

Six-week old wildtype (Wt) and double-transgenic αMHC-CyclinT1/Gαq overexpressing (Cyc) Friend leukemia virus strain B (FVB) mice, kindly provided by M. Schneider and G.W. Dorn, Baylor College of Medicine, Houston, Texas, of either sex were used for this study. In the transgenic mouse model, the heart specific activation of Cdk9 via Cyclin T1 overexpression induces hypertrophy by phosphorylation of the RNA polymerase II. Gαq overexpression as an additional hypertrophic stimulus further enhances transcription elongation. Thus the double transgenic mice show a global increase in RNA synthesis leading to myocyte enlargement and further development of fibrosis as well as apoptosis resulting in heart failure.

### Isolation and cell culture

Adult Sca-1 positive progenitor cells (Sca-1 cells) were isolated from age-matched wildtype (Wt cells) and double-transgenic mice (Cyc cells) as described previously [7]. Briefly, mice were anesthesized in a glass cylinder saturated with inhalant anesthetic isoflurane (4%) and sacrificed via cervical dislocation. Sca-1 cells were isolated from the extracted heart via magnetic cell sorting. Freshly isolated cells or minimally cultured for 4–5 days in DMEM/F12 media (Life Technologies) in 3:1 ratio supplemented with 20% fetal bovine serum (FBS) (Life Technologies), 100units/ml penicillin and 0.1mg/ml streptomycin (Sigma-Aldrich, St. Louis, MO, USA) were used for the study. Cells cultivated for few days, to attain cell adhesion and avoid cell culture-triggered alterations, were considered as undifferentiated cells. Cells isolated from hearts of 2–3 mice were pooled and experiments were performed using three such independent pools (n = 3).

Human umbilical venous endothelial cells (HUVECs) were obtained from collagenase type II (Biochrom, Berlin, Germany) digested umbilical veins and cultivated as described previously [18]. Briefly, umbilical veins were washed thoroughly with sterile phosphate buffered saline (PBS, PAA Laboratories GmbH, Cölbe, Germany) to remove the blood clots and then perfused with 1% collagenase solution. HUVECs were recovered by centrifugation and cultured in MCDB131 media (Pan Biotech, Aidenbach, Germany). For cell migration assays, HUVECs maintained at passage 3 and freshly isolated negatively labeled Sca-1 cells were used as controls.

### RNA isolation and transcriptome profiling

Total RNA was extracted from freshly isolated Cyc and Wt cells of two independent experiments as described previously [7]. Briefly, RNA was extracted using Trizol reagent (Peqlab, Erlangen, Germany) followed by transcriptional profiling with GeneChip Mouse 430 2.0 arrays (Affymetrix, Santa Clara, CA, USA). Then linear amplification was carried out and raw data files were analyzed in Resolver 7.2 (Ceiba Solutions, Seattle, WA, USA). To improve analysis of small sample size experiments, the Rosetta Resolver error model was used for detection of differentially expressed genes [19]. A p-value of <0.05 based on the ratio measurement and its associated error in addition to fold change cut of 2 (Cyc vs. Wt) was considered as significant. Expression data were submitted to GEO.

### *q*RT-PCR

Microarray data were validated by quantitative reverse-transcription polymerase chain reaction (qRT-PCR) using 3 independent biological samples of freshly isolated Sca-1 cells. In addition to selected genes (Bdnf, Ccl19, Ccl9, Crlf1, Cxcl13, Ptn, Sfrp2, Spp1, Wisp2), ß-actin (Actb) was used as endogenous control. PCR amplification was performed using a 7900 HT Sequence Detection System (Applied Biosystems, CA, USA). Thermal cycling was as follows: 2 minutes at 50°C; 10 minutes at 95°C, followed by 15 seconds at 95°C; and 1 minute at 60°C for 45 cycles. Real-time PCR data were quantified using the SDS2.3 software package (Applied Biosystems) followed by relative quantification using the comparative Ct method (ΔΔCt method)[20] (S1 Table).

### Cell Migration Assay

Migration efficiency was determined by using a modified-Boyden chamber containing a nitrocellulose membrane of 8*μ*m pore size (Chemotaxis Assay, Millipore, Schwalbach, Germany). Freshly isolated Sca-1 cells were seeded into the upper chamber at a density of 4x10^4^ cells per well. Subsequently, HUVEC and freshly isolated Sca-1 negative cells were seeded in parallel. Cells were then exposed to different concentrations (0, 10, 25 or 50ng/ml) of BDNF (Santa Cruz Biotechnology, Santa Cruz, CA, USA), which was used as chemoattractant in the lower chamber. Serum-free medium served as negative control and complete medium as background reference. To determine fluorescence at the maximal migration rate, equal numbers of cells were directly seeded to wells of the lower chamber while only medium was added to the upper chamber.

Following 2 hours of incubation at 37°C and 5% CO_2,_ the non-migrated cells were removed from the upper side of the membrane. The cells attached to the lower side of the membrane were retrieved using detachment buffer. Collectively the detached cells and those cells that migrated to the lower chamber were fluorescently labelled with CyQuant dye and measured at 485/530nm. The migrated cells were quantified using the following three variables.

$$\text{Cell migration}\left(\text{\%}\right)=\frac{\text{Mean fluorescence of test - Mean fluorescence of negative control}}{\text{Mean fluorescence of maximum migration}}\times \text{100}$$

### Immunofluorescence

Freshly isolated Sca-1 cells were attached to microscopic slides using a cytocentrifuge (Shandon Cytospin 4, Thermo Scientific, Bremen, Germany) at 100*g* for 8 minutes and afterwards washed with 1x phosphate buffered saline (PBS) to remove non-adherent cells. For cultured cells, Sca-1 cells were allowed to grow on glass coverslips until they reached 70% confluence and subsequently washed with PBS. The adherent cells were then fixed with 4% paraformaldehyde for 10 minutes. After washing with PBS, cells were permeabilized using 4% fetal bovine serum (FBS) and 0.2% Triton in PBS for 30 minutes at room temperature. The cells were then incubated with the primary antibody against TrkB (1:100, Abcam, Cambridge, UK) in 4% FBS and 0.2% Triton in PBS overnight at 4°C. The untreated control was processed in parallel. Cells were washed thoroughly before incubation with the corresponding secondary antibody (goat anti-rabbit Alexa Fluor 488, 1:200 dilution, Life Technologies GmbH, Darmstadt, Germany) for 1 hour at room temperature. Nuclear staining was achieved using DAPI stain (1:100000, Roth, Karlsruhe, Germany). The slides with freshly isolated cells were then visualized using a microscope at 40x magnification while cultured Sca-1 cells were examined at 20x magnification.

### Pulsed SILAC (pSILAC) labeling

In order to perform pulsed SILAC experiments Sca-1 cells had to be cultured up to 70% confluence for 8 days in DMEM/F12 (3:1) medium (Pierce, Thermo Scientific, Bonn, Germany) supplemented with light amino acids ^12^C_6_-L-lysine (120mg/L) and ^12^C_6_-L-arginine (90mg/L) (Sigma-Aldrich, St. Louis, MO, USA), 20% FBS (Biochrom, Berlin, Germany), 3.5mM glutamine, 1 mM sodium pyruvate, 20 mM glucose, 100 units/ml penicillin, and 0.1mg/ml streptomycin (all from Sigma-Aldrich). Afterwards, cells were shifted to medium containing heavy amino acids ^13^C_6_-L-lysine and ^13^C_6_-arginine (Euriso-Top, Saarbruecken, Germany) with or without 25ng/ml BDNF for 24 hours at 37°C. To explore the molecular effects of BDNF, phosphorylation and thereby activation of the TrkB receptor was blocked using the protein kinase inhibitor K252a (Sigma-Aldrich) [21]. For inhibitor treatment, cells were pre-incubated in heavy medium containing 400nM K252a for 2 hours and subsequently washed twice with 1x PBS. Afterwards medium containing heavy amino acids with or without 25ng/ml BDNF was added. After 24 hours of incubation, cells were detached by trypsination and lysed using lysis buffer composed of 8M Urea and 2M Thiourea (Sigma-Aldrich). Three-independent experiments were performed and subjected to quantification by mass spectrometry.

### Sample preparation and mass spectrometric quantification

Cell pellets resuspended in lysis buffer were subjected to 5 cycles of freezing in liquid nitrogen and subsequent rapid thawing at 30°C followed by sonication on ice for 3 seconds with 3-cycles each at 70% energy using a Sonoplus (Bandelin, Berlin, Germany). The homogenates were centrifuged at 16,060*g* for 1 hour at 4°C to spin down insoluble cell debris. The supernatant was collected and protein content was determined using a Bradford Assay kit (Pierce).

For MS analysis, 4*μ*g of each protein sample was digested in-solution followed by reduction with 2.5mM dithiothreitol for 1 hour at 60°C and a subsequent alkylation with 10mM iodoacetamide for 30 minutes in the dark at 37°C. Proteins were digested adding trypsin at a ratio of 1:25 (Promega, Mannheim, Germany) and incubation at 37°C for 16 hours. The reaction was stopped with a final concentration of 1% acetic acid. The resultant peptides were desalted and purified using C-18 reverse phase columns with a binding capacity of 2*μ*g (ZipTip μ-C18, Millipore). MS analysis was performed on a Nano-Acquity UPLC system (Waters Corporation, Milford, MA, USA) connected to a LTQ-Orbitrap-Velos equipped with a nano-ESI source. Extracted peptides were separated using a Nano-Acquity BEH130C18 column (10cm x 100*μ*m, 1.7*μ*m, Waters Corporation) using a 92 minutes non-linear gradient ranging from 1–99% acetonitrile (ACN) in 0.1% acetic acid.

### Processing of MS data

Proteins were identified and quantified using Rosetta Elucidator version 3.3 (Ceiba Solutions). Grouping of labeled pairs was achieved using a PPM error tolerance of 10 ppm, a retention time tolerance of 0.5 minutes and a mass shift of 6.020 Da for both arginine and lysine. The peak lists generated were processed using SEQUEST/ Sorcerer server (Sorcerer version 3.5, Sage-N Research, Milpitas, CA, USA) and searched against a Swiss-Prot mouse database release version 2012/06 with a peptide mass tolerance of 10 ppm and fragment ion tolerance of 1 Da. Oxidation of methionine and SILAC labeling of arginine and lysine were considered as variable modifications while carbamidomethylation of cysteine was regarded as fixed modification. Labeled pairs of proteins designated as good by Elucidator (at least one isotope group in the labeled pair was annotated by a peptide sequence) and identified with a Peptide teller score of at least 0.9 were considered for further analysis. For relative quantification the H/L-ratios, which indicate new protein synthesis against the background of protein present before treatment, were used. Log2 transformed H/L ratios were first median normalized and then ratios between treatment groups and control were calculated. To determine the significance of differences in protein synthesis rates, the statistical t-test implemented in Gene Data Analyst was applied. Only proteins identified with a significance of *p* < 0.05 and a log2 ratio of at least ±0.3 against their respective controls were considered to be differentially expressed between both conditions.

### Cell proliferation assay

Proliferation rate of Sca-1 cells was determined using a Cell Proliferation ELISA, BrdU (colorimetric) Kit (Roche Applied Science, Penzberg, Germany) following the manufacturer’s instructions. Briefly, cells were seeded onto 96-well plates at a density of 10,000 cells/ 100μl/ well in Sca-1 medium and allowed to grow for up to 4 days. The cells were treated with different concentrations of BDNF and incubated for 24 hours. Afterwards, the cells were incubated for 18 hours at 37°C in the presence of 5-Bromo-2’-deoxy-uridine (BrdU, 1:100) which is incorporated during DNA synthesis in replicating cells instead of thymidine and subsequently the cells were fixed by adding FixDenat. The cells were then incubated with anti-BrdU-POD antibody (1:100) for 90 minutes at room temperature followed by washing and addition of substrate. The reaction product was quantified by measuring the absorbance at 490 nm.

### Western Blot analysis

Protein samples of 30 *μ*g each were separated on NuPAGE 4–12% Bis-Tris gels (Life Technologies) according to the manufacturer’s instructions. Following separation, proteins were transferred onto PVDF membranes (Immobilion-P, Millipore) using a Mini Trans-Blot Cell (Bio-Rad Laboratories, Hercules, CA), at a constant voltage of 100 V for 45 minutes. The membranes were blocked with 5% milk in Tris buffered saline (20mM Tris-HCl, 137mM NaCl pH-7.6) containing 0.1% Tween 20 and then incubated with the respective primary antibody overnight at 4°C. The following primary antibodies were used: anti-BDNF, anti-TrkB and anti-phospho TrkB (Abcam); anti-Akt, anti-phospho Akt, anti-p38 MAPK, anti-phospho p38MAPK and anti-ß-actin (Cell signaling, Boston, MA, USA) all raised in rabbit, 1:1000 dilution. Membranes were washed thoroughly before incubation with the secondary antibody conjugated to horseradish peroxidase enzyme at a 1: 10000 dilution for 1 hour. Immunoreactive bands were visualized by chemiluminescence using SuperSignal West Femto maximum sensitivity substrate (Thermo Scientific). Image Quant was applied to quantify the signals of three independent experiments. The relative band intensities were calculated and normalized to a specific experimental control.

### Functional assessment

Gene ontology (GO)-based functional categorization was carried out as described earlier [7]. Briefly, enrichment of differentially regulated genes/proteins was performed using Ingenuity Pathway Analysis (Ingenuity Systems, version 8.6, Redwood City, CA, USA). The significance of the results was evaluated using right-tailed Fisher’s exact test. Downstream effect analysis was performed to determine the top biological functions. The direction of change was predicted based on z-score algorithm [22] with z ≥ 1.5 indicating activation and z ≤ −1.5 inhibition of biological function.

### Statistics

Statistical analyses were performed using one-way ANOVA with multiple comparison test (Newman-Keuls test) or Student’s t-test (paired or unpaired) for pairwise comparisons. Changes in protein abundance were considered to be significantly different at *p* < 0.05. Results are expressed as mean ± SD for at least three independent biological experiments.

## Results

### Transcriptional differences in adult Sca-1 cells from heart failure and normal mice hearts

**Fig 1:**
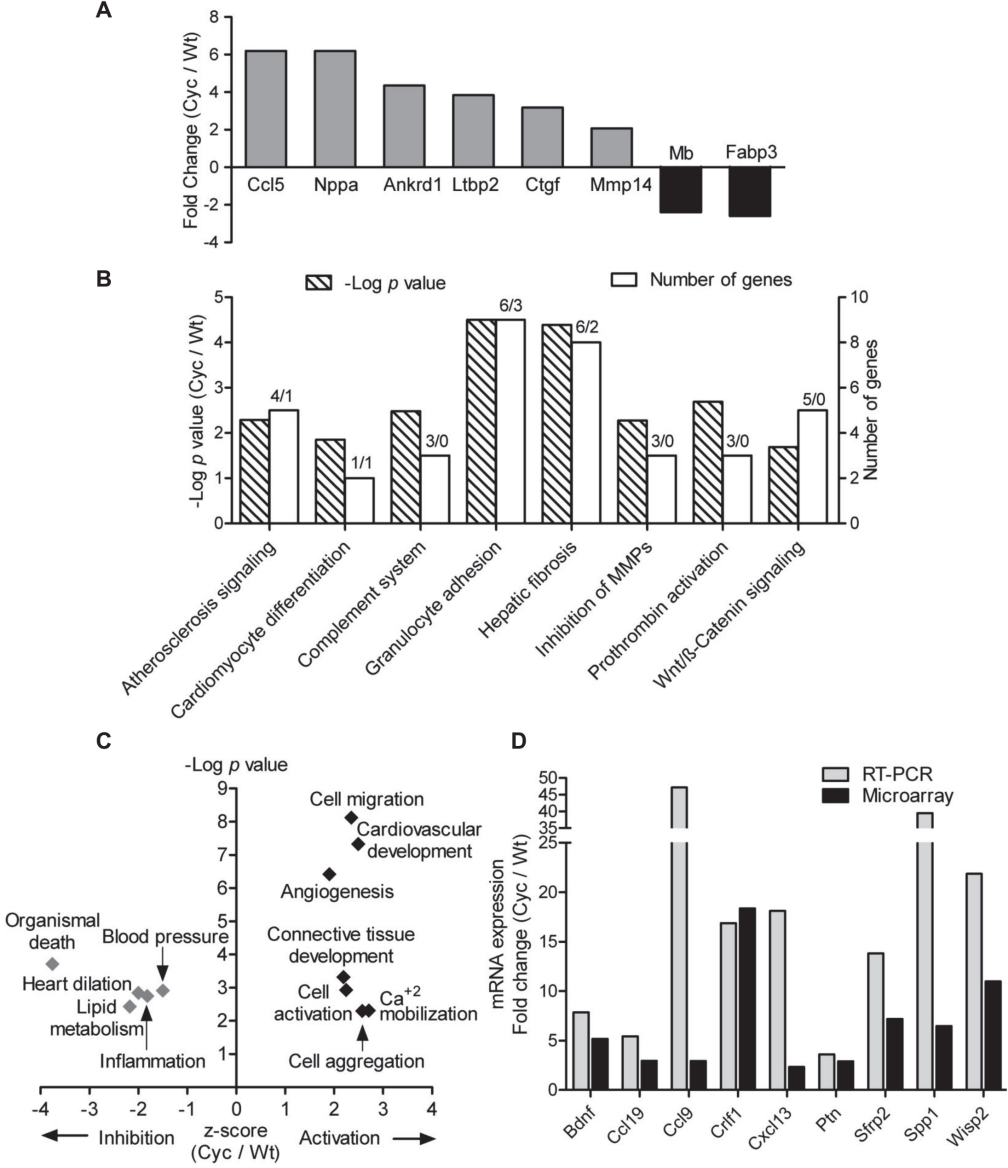
Gene expression alterations in Sca-1 cells in response to heart failure. (A) Relative fold change in expression of genes associated with heart failure in Cyc compared to Wt cells. (B) Top canonical pathways overrepresented by differentially expressed genes in Cyc vs Wt cells.—log *p-*value displays the significance of association dependent of the number of genes in the class calculated by Fisher’s exact test in IPA. Numbers of up-regulated/down-regulated genes are indicated above each bar. (C) Biological functions predicted to be differentially affected based on the differentially expressed genes in Cyc vs Wt cells based on activation z-score calculated in IPA. Black dots denote functional activation (z-score ≥ 1.5) and grey dots functional inhibition (z-score ≤ −1.5). (D) Validation of microarray results by quantitative RT-PCR (n = 3; *p*<0.05; ANOVA).

Double-transgenic αMHC-CyclinT1/Gαq overexpressing mice (Cyc) served as a heart failure model that developed a disease phenotype at the age of 4–6 weeks [23]. This genetically modified mouse model was developed using α-MHC promoter limiting effect of transgenesis to cardiomyocytes while not affecting other heart cell populations (S2 Table). To investigate the changes in response to heart failure at the molecular level, a comparative gene expression analysis of freshly isolated Cyc cells against Wt controls was performed. A total of 197 genes were differentially expressed (*p* < 0.05) with at least a 2-fold difference between Cyc and Wt cells indicating the initiation of a diverse transcriptional program in the pathological milieu. Of these genes, 82% (161/197) were expressed at higher level and only 18% (36/197) displayed lower expression in Cyc than in Wt cells. Also, hypertrophy markers such as natriuretic peptide A (Nppa), chemokine (C-C motif) ligand 5 (Ccl5), connective tissue growth factor (Ctgf), ankyrin repeat domain 1 (Ankrd1) and latent transforming growth factor beta binding protein 2 (Ltbp2) were found to be expressed at higher level in cells from transgenic hearts, while expression of fatty acid binding protein 3 (Fabp3) and myoglobin (Mb) was lower than in cells from Wt hearts as shown in Fig. 1A.

GO-based functional annotation of the differentially expressed genes using IPA software indicated over-representation of pathways for Wnt signaling, cell adhesion, atherosclerosis, prothrombin activation and inhibition of MMPs as shown in Fig. 1B. Enrichment of biological processes predicted activation of cell migration, cardiovascular development, Ca^+2^ mobilization, angiogenesis and inhibition of death, inflammation, and dilation of heart in the progenitor cells from the heart failure model as shown in Fig. 1C, S3 Table. All the differentially expressed genes in both groups of cells were categorized based on IPA analysis into sub-groups such as cytokines, growth factors, enzymes, transcription regulators, and transporters and are listed in S4 Table.

Microarray results were validated using qRT-PCR for nine genes that were expressed at higher level in Cyc compared to Wt cells (Bdnf, Ccl19, Ccl9, Crlf1, Cxcl13, Ptn, Sfrp2, Spp1, and Wisp2). Expression levels of each selected transcript were normalized to the ß-actin control and fold change in expression of target genes in Cyc cells compared to Wt was calculated using the ΔΔCt method. RT-PCR confirmed the microarray data (Fig. 1D) and the reliability of the results.

### Elevation of BDNF expression during heart failure

**Fig 2:**
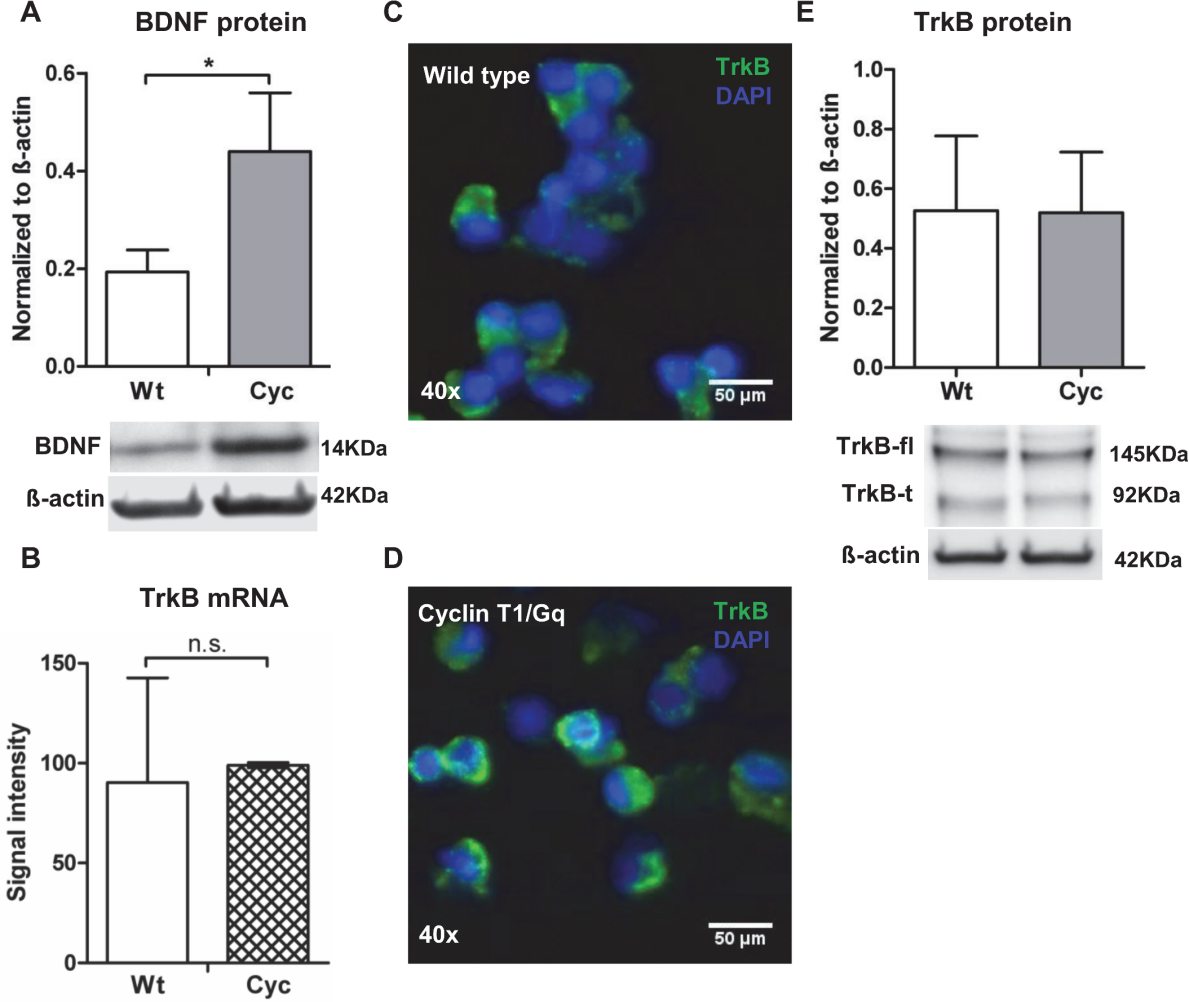
Expression of BDNF and receptor TrkB on progenitor cells. (A) Immunoblot data depicted higher expression of BDNF in freshly isolated Cyc cells in comparison to Wt. Bar graph indicates relative protein abundance of BDNF normalized to ß-actin control quantified by densitometry (n = 3; mean ± SD; *p<0.05; t-test). (B) mRNA intensity signals of TrkB receptor determined by microarray analysis (n = 2; mean ± SD). (C-D) Immunofluorescence micrographs display the expression of TrkB receptor on Sca-1 cells. Nuclei were stained with DAPI. (Scale bar: 50μm, image magnification: 40x). (E) Protein abundance of TrkB receptor on Sca-1 cells quantified against ß-actin control by densitometry (n = 3, mean ± SD). Representative immunoblot of both full length and truncated forms of TrkB receptor on Wt and Cyc cells is shown below.

Although a 5-fold higher expression of the BDNF transcript was observed in Cyc cells, it was important to verify if these differences were represented at the protein level. Immunoblot analysis demonstrated an increase in BDNF protein level in Cyc cells in comparison to Wt (Fig. 2A). Moreover, BDNF is known to mediate its effect via the TrkB receptor, the mRNA of which did not differ between Cyc and Wt cells (Fig. 2B). Immunofluorescence micrographs (Fig. 2C-D) and immunoblots (Fig. 2E) confirmed expression of the BDNF receptor in both groups of cells. However, no significant difference in the abundance of the TrkB receptor was observed between Cyc and Wt cells.

### BDNF improves migration of adult progenitor cells isolated from failing hearts

**Fig 3:**
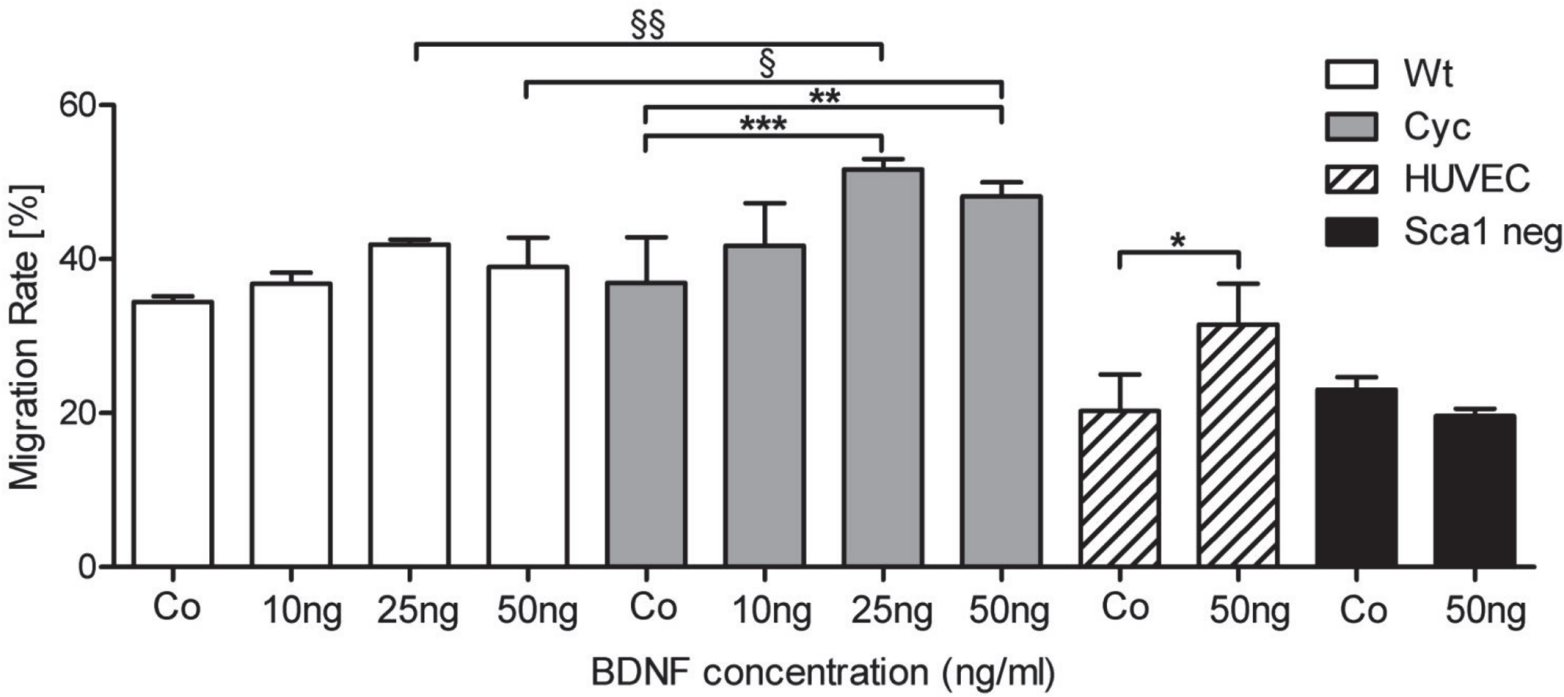
BDNF enhances migration of progenitor cells. Migration of Sca-1 cells (4 x 10^4^) was evaluated using a modified Boyden chamber assay. Freshly isolated cells were exposed to varying concentrations of BDNF (10, 25, 50ng/ml) and allowed to migrate for 2 hours (n = 3; mean ± SD; ANOVA; ***p < 0.0001, **p < 0.001, *p < 0.05 vs Control; §§*p* < 0.001, §*p* < 0.05 vs BDNF).

About 25% of the differentially expressed genes identified by microarray analysis were associated with cell migration (S1 Fig.) indicated by IPA analysis. Higher expression of BDNF in Sca-1 cells derived from failing hearts, prompted us to determine the influence of exogenous BDNF on the migration capacity of undifferentiated Sca-1 cells. BDNF acted as a chemoattractant for Sca-1 cells in a concentration-dependent manner (10, 25 and 50ng/ml), with maximal effect at 25ng/ml (Fig. 3). The significantly higher migration rate (15%) of Cyc cells in comparison to Wt in the presence of 25ng/ml BDNF might indicate an increased migration potential of progenitor cells in the failing heart. HUVECs served as positive control and displayed the expected positive effect of BDNF on migration [24] while freshly isolated Sca-1 negative cells that were used as a negative control due to lack of TrkB receptor, did not display any effect of BDNF on migration (S2 Fig.).

### Quantitation of BDNF induced alterations in protein synthesis

**Fig 4:**
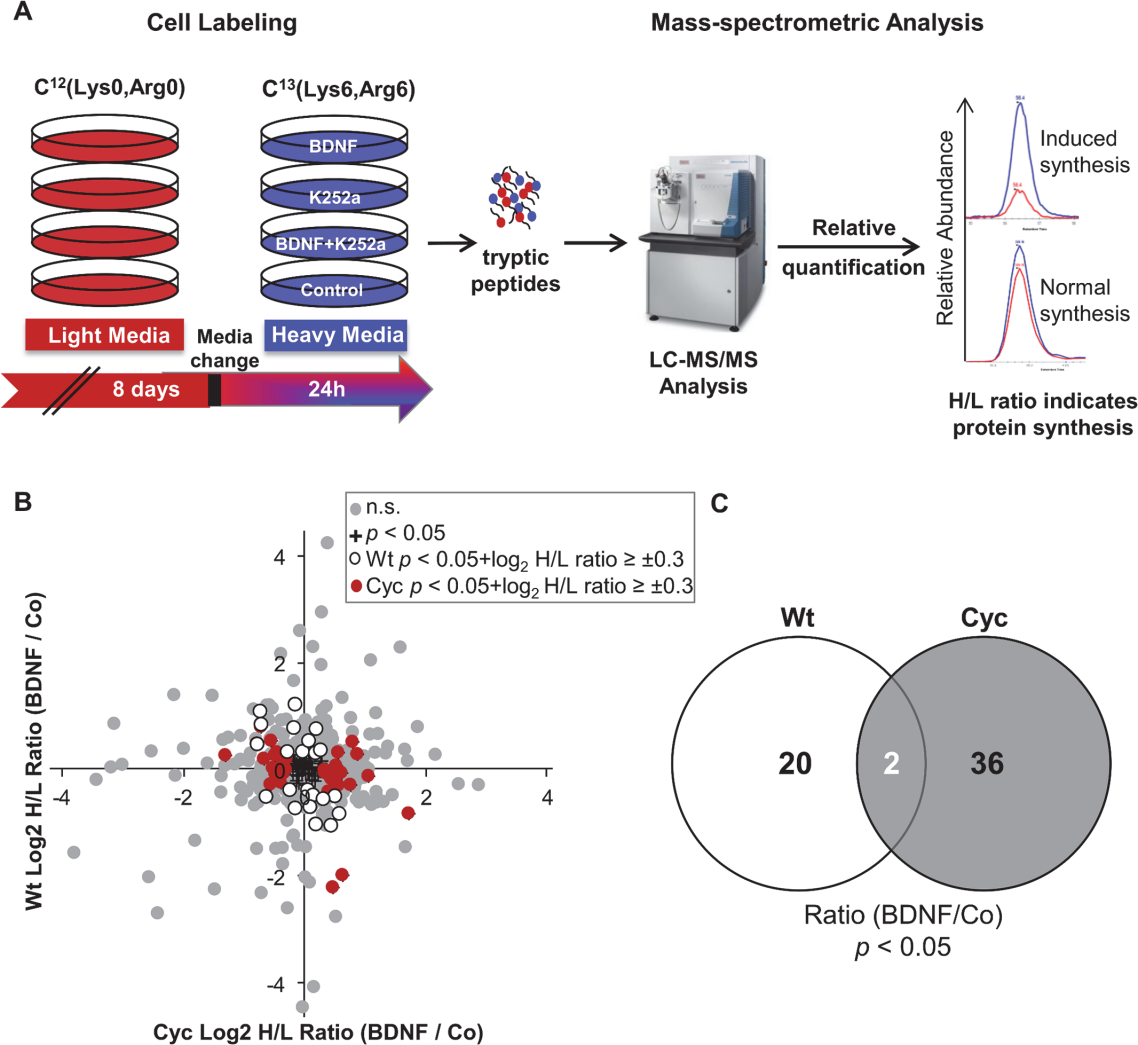
pSILAC analysis of BDNF induced changes in protein synthesis. (A) Schematic representation of pulsed SILAC analysis. Sca-1 cells were cultured for 8days in light medium (red) followed by exchange to medium with heavy lysine and arginine (blue) containing 25ng/ml BDNF or 400nM K252a or a combination of both and allowed to grow for 24 hours. Cell lysates were digested and then subjected to mass spectrometric analysis. Newly synthesized proteins were determined based on H/L ratios. (B) Comparison of BDNF induced changes in the protein synthesis of Cyc and Wt cells (n = 3). Majority of proteins showed moderate change in protein synthesis depicted as grey spots; those identified with statistical significance (*p* < 0.05) are highlighted in black while only differentially regulated proteins in Cyc and Wt cells (*p* < 0.05, log2 ratio ≥ ±0.3) are highlighted in red and white spots respectively. (C) Venn diagram illustrates the overlap of displays protein numbers with altered synthesis rate as a result of BDNF treatment (BDNF vs Co, *p* < 0.05) in both groups of cells.

In order to determine the effect of BDNF on protein synthesis and track the early developmental changes under heart failure condition, pulsed metabolic labeling of Sca-1 cells was carried out as shown in Fig. 4A. To establish stable physiological conditions in the cells after the isolation, cells were cultured in the presence of light amino acid variants for 8 days. TrkB receptor that drives the action of BDNF was also detectable at this time point (S3 Fig.). Afterwards the standard cell culture medium was replaced with medium containing heavy amino acids and cells were simultaneously exposed to different treatments to explore their effect on protein synthesis (pulsed SILAC approach). The different variants included a control, BDNF-treatment (25ng/ml), inhibition of the TrkB receptor with the protein kinase inhibitor K252a and simultaneous addition of the inhibitor K252a and BDNF. For quantitation, the ratios of heavy labeled peptides (synthesized after the medium exchange) to their light labeled counterparts (synthesized prior to the medium change and stable) that are distinguishable based on the peptide mass difference of 6 Da, were used.

Within 24 hours after replacement of light with heavy amino acids, the synthesis of a total of 1650 proteins was observed with high confidence in all triplicate samples. For the majority of these, treatment with either BDNF or K252a had no impact since the log2 ratio did not exceed a threshold of ±0.3 between both conditions and the untreated control (Fig. 4B). In order to identify BDNF induced changes, relative abundance of newly synthesized proteins in BDNF treated Cyc and Wt cells were compared to their respective controls. Inhibiting the phosphorylation of TrkB receptor by K252a treatment (S4A Fig.) should alleviate the effects of BDNF and thus proof the specificity of the BDNF effect (S4B Fig.)[25]. In comparison to untreated controls BDNF treatment triggered significant difference (*p* <0.05) in the fraction of newly synthesized proteins for 115 proteins in either the Wt or Cyc cells and for 58 of those proteins the difference was larger than 1.23 fold (log_2_ ratio of at least ±0.3, Fig. 4B). The Venn diagram displayed in Fig. 4C illustrates that BDNF basically targeted entirely different protein populations in Cyc and Wt cells with only a minimal overlap. The two proteins regulated upon BDNF treatment in Cyc and Wt cells-SRRT and PSME2 showed an inverse regulation pattern.

**Fig 5:**
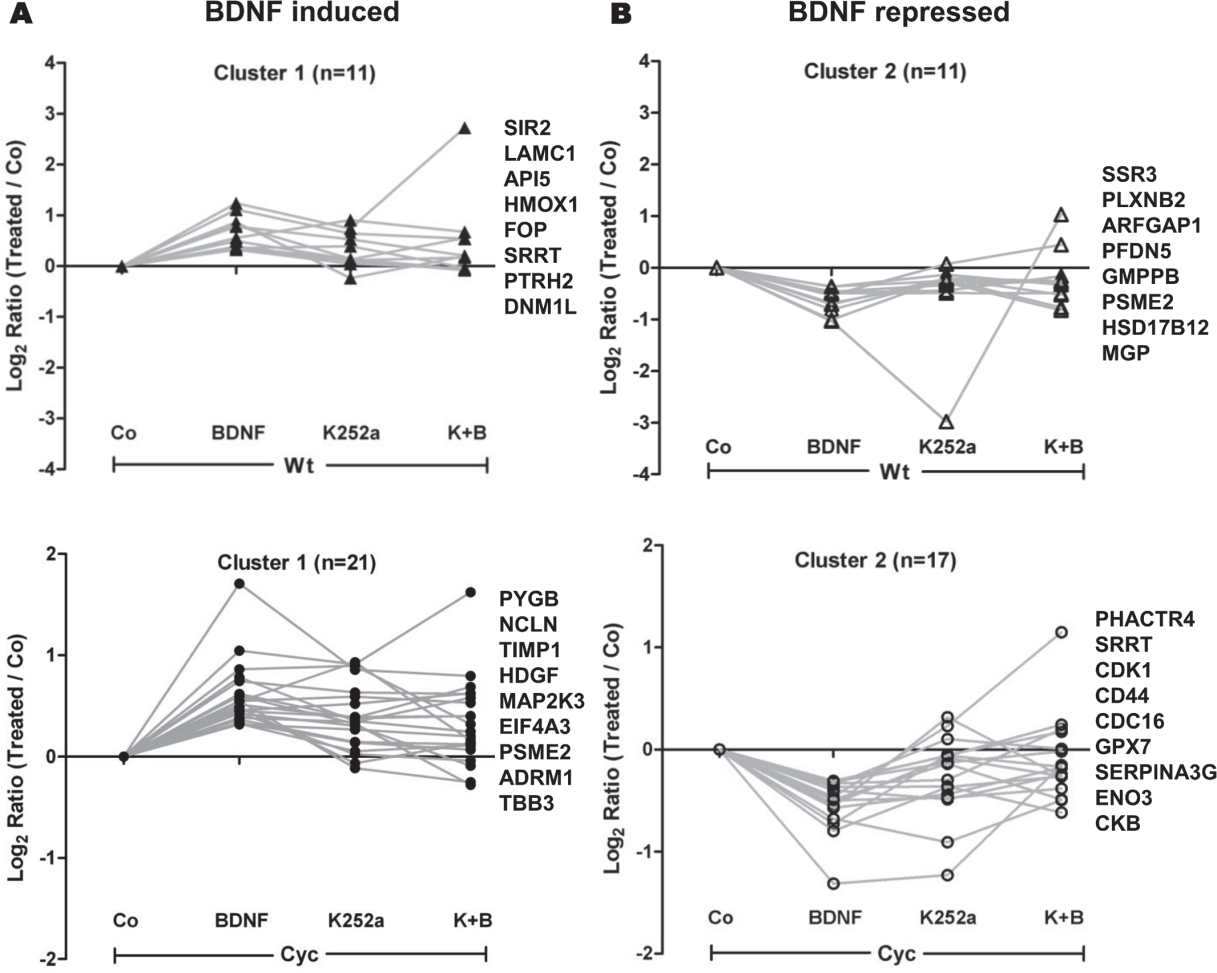
K-means clustering of newly synthesized proteins. K-means clustering of differentially regulated proteins under different treatment conditions compared to their respective controls. (A) represents BDNF induced up-regulation in protein synthesis, while (B) represents down regulation. Top regulated proteins in response to BDNF treatment are specified in each cluster (S5 Table *)*.

A more detailed analysis of regulated proteins was carried out using K-means clustering based on positive correlation. BDNF-regulated proteins of cells from Cyc and Wt hearts were assigned to 2 clusters representing positive (Cluster 1) and negative (Cluster 2) effects of BDNF, respectively. The log2 ratios of the different treatment conditions of the top regulated proteins of each cluster are specified in Fig. 5, S5 Table. Furthermore, functional annotation of these regulated proteins using IPA analysis revealed BDNF induced synthesis of cell survival proteins such as API5, HMOX1 and SIR2 in Wt cells. In addition, proteins associated with cell proliferation like LAMC1, SRRT, DNM1L were induced in these cells. Other proteins that negatively regulated cell proliferation such as PLXNB2 and MGP were repressed in Wt cells as a result of BDNF stimulation. However, in Cyc cells BDNF induced synthesis of cell survival proteins such as HDGF, TIMP1 and TUBB3, while proteins associated with cell proliferation such as CDK1, CDC16, SRRT and PHACTR4 were repressed suggesting a reduction of mitotic events under pathological conditions.

### BDNF-mediated effects on Sca-1 cell proliferation

**Fig 6:**
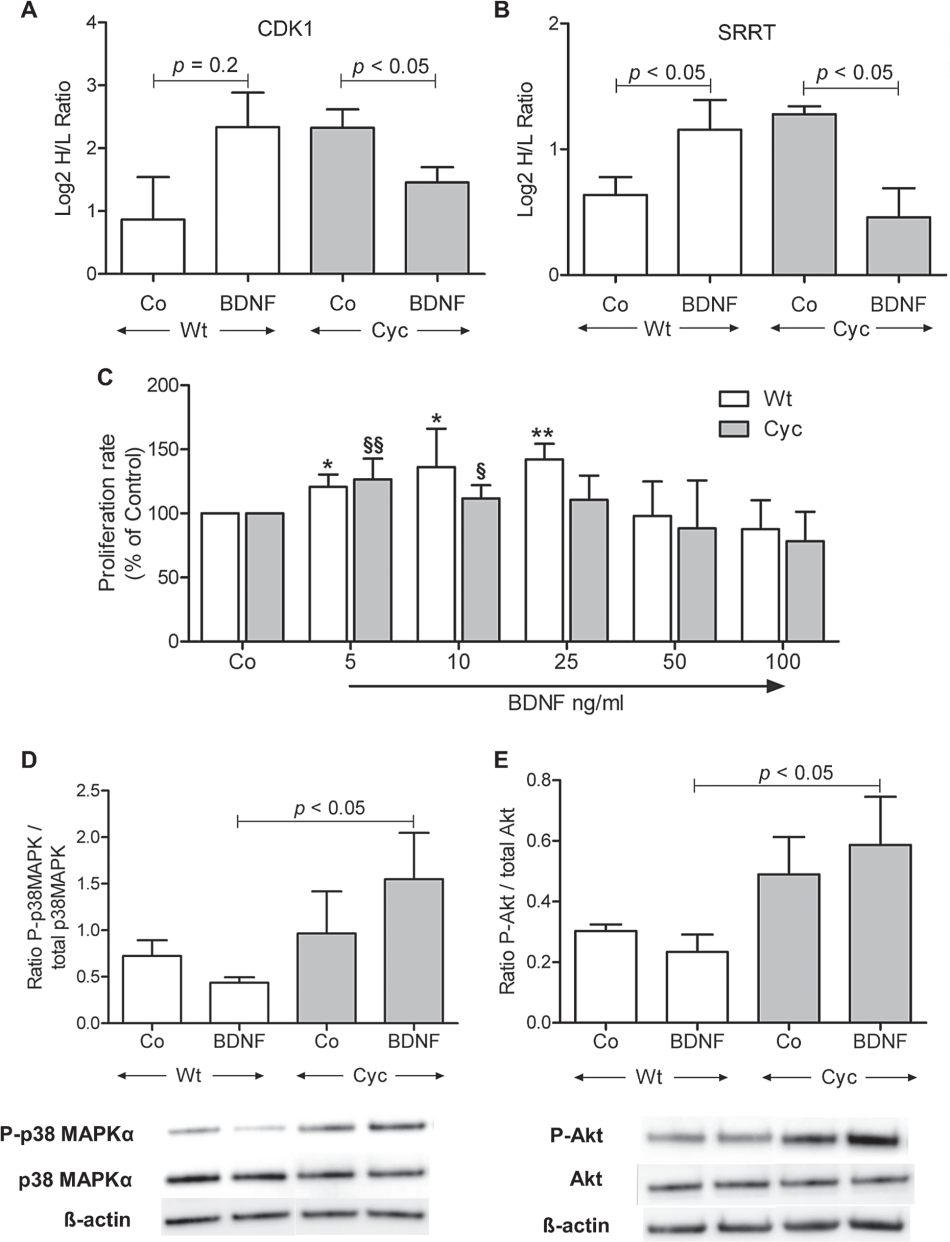
BDNF-mediated alterations on Sca-1 cells. (A, B) Relative changes in protein synthesis of CDK1 and SRRT denoted as log2 H/L ratio for Wt and Cyc cells under BDNF treated and untreated conditions (n = 3; mean ± SD; * *p* < 0.05; t-test). (C) Impact of BDNF on cell proliferation of Sca-1cells as assessed by BrdU incorporation after 24 hours treatment. Change in proliferation rate in BDNF treated cells was determined in comparison to the untreated condition in respective cells (n = 3; mean ± SD; * *p* < 0.05, ** *p* < 0.01 against Wt Co, § *p* < 0.05, §§ *p*<0.01 against Cyc Co; t-test, paired). (D, E) Representative immunoblots indicating the degree of phosphorylation of p38 MAPKα (D) and Akt (E). Quantification of phosphorylation was achieved by normalization against band intensities of the unphosphorylated form (n = 3; mean ± SD; * *p* <0.05; ANOVA).

Opposite effects of BDNF were observed by the pSILAC analysis for proteins associated with cell cycle progression such as CDK1 and SRRT (Fig. 6A,B) in Sca-1 cells originating from control or transgenic hearts. While BDNF stimulated the synthesis of both proteins in Wt cells, their synthesis was decreased in Cyc cells. Therefore, we further assessed the effect of BDNF on the proliferative potential of Sca-1 cells using a BrdU-based assay in which proliferation rate was determined by comparing BrdU incorporation in BDNF-stimulated versus untreated control cells. BDNF stimulated the proliferation of Wt cells up to 25ng/ml BDNF in a concentration-dependent manner while such an effect was not seen with Cyc cells (Fig. 6C). The proliferative capacity of Cyc and Wt untreated control cells did not differ significantly (1.1 fold; *p* < 0.1, Student’s t-test). In addition, immunoblots revealed that BDNF induced activation of p38MAPK signaling in Cyc cells while a reverse effect was observed in Wt cells (Fig. 6D). Since the p38MAPK signal is reported to activate cell differentiation processes by inhibiting cell proliferation [26], our results might provide an indication of BDNF treatment induced reduction in cell cycle progression in Cyc cells probably leading to initiation of cell differentiation.

Furthermore BDNF treatment slightly improved phosphorylation of Akt (Fig. 6E) in Cyc cells but decreased the same in Wt cells. Under BDNF-stimulation Akt-phosphorylation was significantly stronger in Cyc cells compared to Wt, suggesting an improved pro-survival effect of BDNF under pathological conditions. However, no significant difference was observed in the absence of BDNF stimulation. Hence, immunoblot results further supported the findings of pSILAC analysis.

## Discussion

The adult mammalian heart harbors a subpopulation of cardiac progenitor cells that are capable of restoring myocardial function after injury. Homing of stem cells to a heart lesion is one of the pre-requisites for inducing repair processes via stem cell transplantation. Oh *et al*. have previously reported that resident Sca-1 cells home to the damaged myocardium and differentiate in part into functional cardiomyocytes when injected into diseased animals but showed no effect in healthy animals [4]. Therefore, a better understanding of molecular events during disease is quite essential for designing effective treatment options. To our knowledge the present study is the first to analyze the molecular characteristics of adult resident Sca-1 positive progenitor cells derived from the failing heart.

To delineate the heart failure mediated molecular changes, we first performed a microarray-based transcriptome profiling of freshly isolated undifferentiated Sca-1 cells derived from failing hearts in comparison to their healthy counterparts. In general transgenic Cyc cells as well as healthy Wt cells shared a similar expression profile. A subset of 197genes showed differential expression indicating initiation of a specific expression program in response to heart failure. Thus, in Cyc cells we observed higher expression of genes encoding Nppa, Ccl5, Ctgf and Ankrd1, which have been shown to be associated with heart failure in earlier studies [27–30]. Furthermore, functional allocation of differentially expressed genes revealed higher enrichment of GO categories such as cell migration, angiogenesis, cardiovascular development, and Ca^+2^ mobilization suggesting the development of cardiogenic repair potential following injury. A large proportion (23%) of the differentially expressed genes was associated with cell migration, of which Bdnf, Ccl5, Postn, Ptn were among the top candidates [31–33]. Genes belonging to ECM such as Col1a1, Ltbp2, Ctgf, Postn, Bgn, Mmp14 and Mmp23b were strongly expressed in Cyc cells suggesting their potential to orchestrate in the cardiac healing process under pathological conditions [34]. Moreover, pathway analysis depicted enrichment of genes of the Wnt/ß-catenin signaling cascade (Sfrp2, Frzb, Dkk3, Fzd2, Cdh2), which represent important regulators of stem cell renewal and differentiation [35]. Additionally Notch3, Irf7 and Celf4 transcription factors were highly expressed in Cyc cells which are known to play a crucial role in promoting protection against cardiac fibrosis in response to injury [36,37]. Overall, these results suggest that Sca-1 cells exhibit a tightly regulated mechanism that controls stem cell renewal and homeostasis probably leading to healing of the injured heart.

Besides showing higher BDNF mRNA amounts, the level of BDNF protein was also higher in Cyc compared to Wt cells. Recently Okada *et al*. have reported encouraging results on restoration of cardiac function post-myocardial infarction, after peripheral administration of BDNF [14]. In addition to the paracrine effect of BDNF on major heart cell populations such as cardiomyocytes or fibroblasts, there might exist an autocrine effect. Here, we have shown that Sca-1 cells express the TrkB receptor, which prompted us to investigate the autocrine effect of exogenously added BDNF on progenitor cells. Assessment of the migratory potential of Sca-1 cells revealed no difference between Sca-1 cells of Cyc and Wt hearts under control conditions, which indicates that endogenous BDNF does not affect cell migration. However, exogenous addition of BDNF significantly improved the migration rate in a dose dependent manner in Cyc cells, with a maximum effect at 25ng/ml. HUVECs showed a similar increase in migration potential in the presence of BDNF. Hence our study suggests that the mobilization of adult Sca-1 cells derived from damaged myocardium is facilitated in the presence of BDNF, which in turn might improve their homing efficiency. However investigation on the paracrine effect of BDNF is currently ongoing.

Although BDNF was shown to positively regulate pro-survival and pro-angiogenic effects on neural stem cells [38], its biological activity is not solely limited to the brain. We aimed to determine the regulatory effect of this neurotrophic factor on cardiac progenitor cells, which are known to attenuate heart dysfunction [13]. Mass spectrometry-based proteomics offers the opportunity to gain insight into cellular physiology [39]. Previous works have employed SILAC strategies to determine the protein turnover using differential labeling with amino acid variants [40]. Schwanhaeusser *et al*. have developed a pSILAC approach [16] that would allow direct quantification of endogenous protein translation in response to external stimuli. Most of the proteins involved in signal transduction and transcriptional regulation are present in low abundance and are dramatically affected in response to perturbation. Such small changes in total protein level are difficult to detect against a large background of preformed proteins. Therefore, we have implemented a slightly modified pSILAC technique using heavy and light variants of amino acids to increase the sensitivity of detecting changes in the protein profile that results from a response to the BDNF stimulus. Such a pulsed labeling approach would focus on tracing the newly synthesized proteins after the addition of BDNF, which might be beneficial to determine the fate of the investigated precursor cells. The global protein synthesis profile of Cyc or Wt cells was not altered by the addition of either BDNF or the TrkB inhibitor K252a alone or in combination. Of 1650 proteins monitored only 58 proteins were differentially regulated in Cyc and Wt cells upon BDNF stimulation. In addition, the effect of BDNF on protein synthesis in Sca-1 cells from Wt and Cyc hearts largely differed, indicating a divergent effect of BDNF in Sca-1 cells under physiological and pathological conditions.

In an attempt to explore BDNF-mediated regulation in Sca-1 cells, all differentially regulated proteins were grouped into clusters representing positive or negative BDNF-effects, respectively. Functional annotation of each cluster indicated enrichment of categories such as cell survival and cell cycle progression. Protein synthesis of HDGF, TIMP1 and TUBB3, well known inhibitors of cell death, [41,42] was up regulated in Cyc stimulated cells while other survival proteins such as API5, HMOX1, SIR2 were up regulated in Wt cells [43,44]. Moreover, Western blot analysis illustrated increased phosphorylation of Akt which is the major mediator of survival signaling [45]. Hence, exogenous BDNF stimulation might be in part responsible for survival of progenitor cells derived from failing hearts [11,46]. In addition to that, we observed contrasting regulation patterns in both cell types, e.g., proteins that drive cell cycle progression such as CDK1and SRRT were down regulated in Cyc cells while they were shown to be up regulated in Wt cells [25,47]. BDNF stimulated the proliferation of Wt cells while the proliferation rate decreased in Cyc cells after 24 hours treatment that further supported the findings of pSILAC analysis. Besides up regulation of MAP2K3 (S5 Fig.) indicated in pSILAC data, an increased phosphorylation of p38MAPK, which is a downstream target of MAP2K3, probably indicated the initiation of a differentiation program by suppressing mitotic events in Cyc cells [26,48].

## Conclusion

Here we have generated a comprehensive overview of global molecular changes in adult Sca-1 cells underlying heart failure conditions. Our comparative microarray-based study provided insights into their endogenous behavior during heart failure and unraveled their potential characteristics. The results indicate that the cardiogenic potential of Sca-1 cells is enhanced during heart failure condition. In addition, we explored the functional implications of BDNF expression, which was found to be elevated in failing hearts. We further provided supporting evidence on the regulatory role of exogenous BDNF on migration, proliferation and survival of progenitor cells, which might offer a therapeutic option for treatment of heart failure. However, it remains to be investigated whether local delivery of BDNF will improve the functional role of Sca-1 progenitor cells inside the damaged myocardium. Further *in vivo* experiments are necessary to extend our knowledge on BDNF driven fate of adult Sca-1 cells in cardiac dysfunction.

## Supporting Information

S1 Fig Gene expression profile of cell migration associated genes in Cyc and Wt cells. (TIF)

S2 Fig Immunoblot analysis of TrkB receptor in Sca-1 positive and Sca-1 negative cells. (TIF)

S3 Fig Expression of TrkB receptor on cultured cells. (TIF)

S4 Fig Blocking of TrkB receptor. (TIF)

S5 Fig BDNF regulates the synthesis of MAP2K3. (TIF)

S1 Table List of Taqman genes expression assays used in qRT PCR analysis. (DOCX)

S2 Table Gene expression of Cyclin T1 (Ccnt1), Gαq (Gnaq) and Sca-1 (Ly6a) in Sca-1 cells derived from transgenic and wildtype control mice. (DOCX)

S3 Table Functional enrichment of biological processes in Cyc cells compared to Wt cells. (DOCX)

S4 Table List of differentially expressed genes in Cyc cells compared to Wt cells. (DOCX)

S5 Table K-means clustering of BDNF- mediated regulated proteins in Cyc and Wt cells. Data represents average of log2 transformed H/L ratios.
(DOCX)

## References

[1] Bernstein HS, Srivastava D (2012) Stem cell therapy for cardiac disease. Pediatr Res 71: 491–499. 10.1038/pr.2011.61 22430385

[2] Garbern JC, Lee RT (2013) Cardiac stem cell therapy and the promise of heart regeneration. Cell Stem Cell 12: 689–698. 10.1016/j.stem.2013.05.008 23746978 PMC3756309

[3] Anversa P, Kajstura J, Rota M, Leri A (2013) Regenerating new heart with stem cells. J Clin Invest 123: 62–70. 10.1172/JCI63068 23281411 PMC3533279

[4] Oh H, Bradfute SB, Gallardo TD, Nakamura T, Gaussin V, Mishina Y, et al (2003) Cardiac progenitor cells from adult myocardium: homing, differentiation, and fusion after infarction. Proc Natl Acad Sci U S A 100: 12313–12318. 14530411 10.1073/pnas.2132126100 PMC218755

[5] Bradfute SB, Graubert TA, Goodell MA (2005) Roles of Sca-1 in hematopoietic stem/progenitor cell function. Exp Hematol 33: 836–843. 15963860 10.1016/j.exphem.2005.04.001

[6] Tateishi K, Ashihara E, Takehara N, Nomura T, Honsho S, Nakagami T, et al (2007) Clonally amplified cardiac stem cells are regulated by Sca-1 signaling for efficient cardiovascular regeneration. J Cell Sci 120: 1791–1800. 17502484 10.1242/jcs.006122

[7] Samal R, Ameling S, Wenzel K, Dhople V, Volker U, Felix SB, et al (2012) OMICS-based exploration of the molecular phenotype of resident cardiac progenitor cells from adult murine heart. J Proteomics 75: 5304–5315. 10.1016/j.jprot.2012.06.010 22749858

[8] Chang HY, Thomson JA, Chen X (2006) Microarray analysis of stem cells and differentiation. Methods Enzymol 420: 225–254. 17161699 10.1016/S0076-6879(06)20010-7

[9] Dey D, Han L, Bauer M, Sanada F, Oikonomopoulos A, Hosoda T, et al (2013) Dissecting the molecular relationship among various cardiogenic progenitor cells. Circ Res 112: 1253–1262. 10.1161/CIRCRESAHA.112.300779 23463815 PMC3657513

[10] Chen BY, Wang X, Wang ZY, Wang YZ, Chen LW, Luo ZJ (2013) Brain-derived neurotrophic factor stimulates proliferation and differentiation of neural stem cells, possibly by triggering the Wnt/beta-catenin signaling pathway. J Neurosci Res 91: 30–41. 10.1002/jnr.23138 23023811

[11] Kim H, Li Q, Hempstead BL, Madri JA (2004) Paracrine and autocrine functions of brain-derived neurotrophic factor (BDNF) and nerve growth factor (NGF) in brain-derived endothelial cells. J Biol Chem 279: 33538–33546. 15169782 10.1074/jbc.M404115200

[12] Caporali A, Emanueli C (2009) Cardiovascular actions of neurotrophins. Physiol Rev 89: 279–308. 10.1152/physrev.00007.2008 19126759 PMC2836529

[13] Leenen FH, Tuana BS (2012) Cardioprotective brain mechanisms. Arterioscler Thromb Vasc Biol 32: 1749–1750. 10.1161/ATVBAHA.112.252627 22815337

[14] Okada S, Yokoyama M, Toko H, Tateno K, Moriya J, Shimizu I, et al (2012) Brain-derived neurotrophic factor protects against cardiac dysfunction after myocardial infarction via a central nervous system-mediated pathway. Arterioscler Thromb Vasc Biol 32: 1902–1909. 10.1161/ATVBAHA.112.248930 22556331

[15] Kermani P, Rafii D, Jin DK, Whitlock P, Schaffer W, Chiang A, et al (2005) Neurotrophins promote revascularization by local recruitment of TrkB+ endothelial cells and systemic mobilization of hematopoietic progenitors. J Clin Invest 115: 653–663. 15765148 10.1172/JCI200522655 PMC1051987

[16] Schwanhausser B, Gossen M, Dittmar G, Selbach M (2009) Global analysis of cellular protein translation by pulsed SILAC. Proteomics 9: 205–209. 10.1002/pmic.200800275 19053139

[17] Mann M (2006) Functional and quantitative proteomics using SILAC. Nat Rev Mol Cell Biol 7: 952–958. 17139335 10.1038/nrm2067

[18] Landsberger M, Wolff B, Jantzen F, Rosenstengel C, Vogelgesang D, Staudt A, et al (2007) Cerivastatin reduces cytokine-induced surface expression of ICAM-1 via increased shedding in human endothelial cells. Atherosclerosis 190: 43–52. 16529752 10.1016/j.atherosclerosis.2006.02.009

[19] Weng L, Dai H, Zhan Y, He Y, Stepaniants SB, Bassett DE (2006) Rosetta error model for gene expression analysis. Bioinformatics 22: 1111–1121. 16522673 10.1093/bioinformatics/btl045

[20] Livak KJ, Schmittgen TD (2001) Analysis of relative gene expression data using real-time quantitative PCR and the 2(-Delta Delta C(T)) Method. Methods 25: 402–408. 11846609 10.1006/meth.2001.1262

[21] Lecht S, Arien-Zakay H, Kohan M, Lelkes PI, Lazarovici P (2010) Angiostatic effects of K252a, a Trk inhibitor, in murine brain capillary endothelial cells. Mol Cell Biochem 339: 201–213. 10.1007/s11010-010-0386-9 20148355

[22] Viljoen KS, Blackburn JM (2013) Quality assessment and data handling methods for Affymetrix Gene 1.0 ST arrays with variable RNA integrity. BMC Genomics 14: 14 10.1186/1471-2164-14-14 23324084 PMC3557148

[23] Sano M, Wang SC, Shirai M, Scaglia F, Xie M, Sakai S, et al (2004) Activation of cardiac Cdk9 represses PGC-1 and confers a predisposition to heart failure. Embo J 23: 3559–3569. 15297879 10.1038/sj.emboj.7600351 PMC516624

[24] Matsuda S, Fujita T, Kajiya M, Takeda K, Shiba H, Kawaguchi H, et al (2012) Brain-derived neurotrophic factor induces migration of endothelial cells through a TrkB-ERK-integrin alphaVbeta3-FAK cascade. J Cell Physiol 227: 2123–2129. 10.1002/jcp.22942 21769870

[25] Ovejero-Benito MC, Frade JM (2013) Brain-derived neurotrophic factor-dependent cdk1 inhibition prevents G2/M progression in differentiating tetraploid neurons. PLoS ONE 8: e64890 10.1371/journal.pone.0064890 23741412 PMC3669015

[26] Engel FB, Schebesta M, Duong MT, Lu G, Ren S, Madwed JB, et al (2005) p38 MAP kinase inhibition enables proliferation of adult mammalian cardiomyocytes. Genes Dev 19: 1175–1187. 15870258 10.1101/gad.1306705 PMC1132004

[27] Horsthuis T, Houweling AC, Habets PE, de Lange FJ, el Azzouzi H, Clout DE, et al (2008) Distinct regulation of developmental and heart disease-induced atrial natriuretic factor expression by two separate distal sequences. Circ Res 102: 849–859. 10.1161/CIRCRESAHA.107.170571 18276916

[28] Montecucco F, Braunersreuther V, Lenglet S, Delattre BM, Pelli G, Buatois V, et al (2012) CC chemokine CCL5 plays a central role impacting infarct size and post-infarction heart failure in mice. Eur Heart J 33: 1964–1974. 10.1093/eurheartj/ehr127 21606075

[29] Panek AN, Posch MG, Alenina N, Ghadge SK, Erdmann B, Popova E, et al (2009) Connective tissue growth factor overexpression in cardiomyocytes promotes cardiac hypertrophy and protection against pressure overload. PLoS ONE 4: e6743 10.1371/journal.pone.0006743 19707545 PMC2727794

[30] Duboscq-Bidot L, Charron P, Ruppert V, Fauchier L, Richter A, Tavazzi L, et al (2009) Mutations in the ANKRD1 gene encoding CARP are responsible for human dilated cardiomyopathy. Eur Heart J 30: 2128–2136. 10.1093/eurheartj/ehp225 19525294

[31] Long H, Xie R, Xiang T, Zhao Z, Lin S, Liang Z, et al (2012) Autocrine CCL5 signaling promotes invasion and migration of CD133+ ovarian cancer stem-like cells via NF-kappaB-mediated MMP-9 upregulation. Stem Cells 30: 2309–2319. 10.1002/stem.1194 22887854

[32] Li G, Jin R, Norris RA, Zhang L, Yu S, Wu F, et al (2010) Periostin mediates vascular smooth muscle cell migration through the integrins alphavbeta3 and alphavbeta5 and focal adhesion kinase (FAK) pathway. Atherosclerosis 208: 358–365. 10.1016/j.atherosclerosis.2009.07.046 19695571 PMC2841688

[33] Heiss C, Wong ML, Block VI, Lao D, Real WM, Yeghiazarians Y, et al (2008) Pleiotrophin induces nitric oxide dependent migration of endothelial progenitor cells. J Cell Physiol 215: 366–373. 17960557 10.1002/jcp.21313 PMC2697332

[34] Hutchinson KR, Stewart JA Jr, Lucchesi PA (2010) Extracellular matrix remodeling during the progression of volume overload-induced heart failure. J Mol Cell Cardiol 48: 564–569. 10.1016/j.yjmcc.2009.06.001 19524591 PMC2824070

[35] Nusse R, Fuerer C, Ching W, Harnish K, Logan C, Zeng A, et al (2008) Wnt signaling and stem cell control. Cold Spring Harb Symp Quant Biol 73: 59–66. 10.1101/sqb.2008.73.035 19028988

[36] Nemir M, Metrich M, Plaisance I, Lepore M, Cruchet S, Berthonneche C, et al. (2012) The Notch pathway controls fibrotic and regenerative repair in the adult heart. Eur Heart J. 10.1093/eurheartj/ehs269 PMC4139705 23166366

[37] Ladd AN, Taffet G, Hartley C, Kearney DL, Cooper TA (2005) Cardiac tissue-specific repression of CELF activity disrupts alternative splicing and causes cardiomyopathy. Mol Cell Biol 25: 6267–6278. 15988035 10.1128/MCB.25.14.6267-6278.2005 PMC1168813

[38] Kingham PJ, Kolar MK, Novikova LN, Novikov LN, Wiberg M (2013) Stimulating the neurotrophic and angiogenic properties of human adipose derived stem cells enhances nerve repair. Stem Cells Dev. 10.1089/scd.2013.0396 24124760

[39] Arab S, Gramolini AO, Ping P, Kislinger T, Stanley B, van Eyk J, et al (2006) Cardiovascular proteomics: tools to develop novel biomarkers and potential applications. J Am Coll Cardiol 48: 1733–1741. 17084242 10.1016/j.jacc.2006.06.063

[40] Boisvert FM, Ahmad Y, Gierlinski M, Charriere F, Lamont D, Scott M, et al (2012) A quantitative spatial proteomics analysis of proteome turnover in human cells. Mol Cell Proteomics 11: M111 011429. 10.1074/mcp.M111.011429 PMC3316722 21937730

[41] Tsang TY, Tang WY, Tsang WP, Co NN, Kong SK, Kwok TT (2008) Downregulation of hepatoma-derived growth factor activates the Bad-mediated apoptotic pathway in human cancer cells. Apoptosis 13: 1135–1147. 10.1007/s10495-008-0241-6 18651222

[42] Guo LJ, Luo XH, Xie H, Zhou HD, Yuan LQ, Wang M, et al (2006) Tissue inhibitor of matrix metalloproteinase-1 suppresses apoptosis of mouse bone marrow stromal cell line MBA-1. Calcif Tissue Int 78: 285–292. 16691494 10.1007/s00223-005-0092-x

[43] Cai C, Teng L, Vu D, He JQ, Guo Y, Li Q, et al (2012) The heme oxygenase 1 inducer (CoPP) protects human cardiac stem cells against apoptosis through activation of the extracellular signal-regulated kinase (ERK)/NRF2 signaling pathway and cytokine release. J Biol Chem 287: 33720–33732. 22879597 10.1074/jbc.M112.385542 PMC3460469

[44] Wang H, Liu H, Chen K, Xiao J, He K, Zhang J, et al (2012) SIRT1 promotes tumorigenesis of hepatocellular carcinoma through PI3K/PTEN/AKT signaling. Oncol Rep 28: 311–318. 10.3892/or.2012.1788 22552445

[45] Guo S, Som AT, Waeber C, Lo EH (2012) Vascular neuroprotection via TrkB- and Akt-dependent cell survival signaling. J Neurochem 123 Suppl 2: 58–64. 10.1111/j.1471-4159.2012.07944.x 23050643 PMC3503457

[46] Ji R, Meng L, Jiang X, Cvm NK, Ding J, Li Q, et al (2014) TAM Receptors support neural stem cell survival, proliferation and neuronal differentiation. PLoS One 9: e115140 10.1371/journal.pone.0115140 25514676 PMC4267817

[47] Diril MK, Ratnacaram CK, Padmakumar VC, Du T, Wasser M, Coppola V, et al (2012) Cyclin-dependent kinase 1 (Cdk1) is essential for cell division and suppression of DNA re-replication but not for liver regeneration. Proc Natl Acad Sci U S A 109: 3826–3831. 10.1073/pnas.1115201109 22355113 PMC3309725

[48] Hadjal Y, Hadadeh O, Yazidi CE, Barruet E, Binetruy B (2013) A p38MAPK-p53 cascade regulates mesodermal differentiation and neurogenesis of embryonic stem cells. Cell Death Dis 4: e737 10.1038/cddis.2013.246 23887628 PMC3730419

