# Supplementary figures and images for: Brain Derived Neurotrophic Factor Contributes to the Cardiogenic Potential of Adult Resident Progenitor Cells in Failing Murine Heart
Source: PLoS One. 2015 Mar 23;10(3):e0120360. doi: 10.1371/journal.pone.0120360 (PMC4370398; doi:10.1371/journal.pone.0120360)

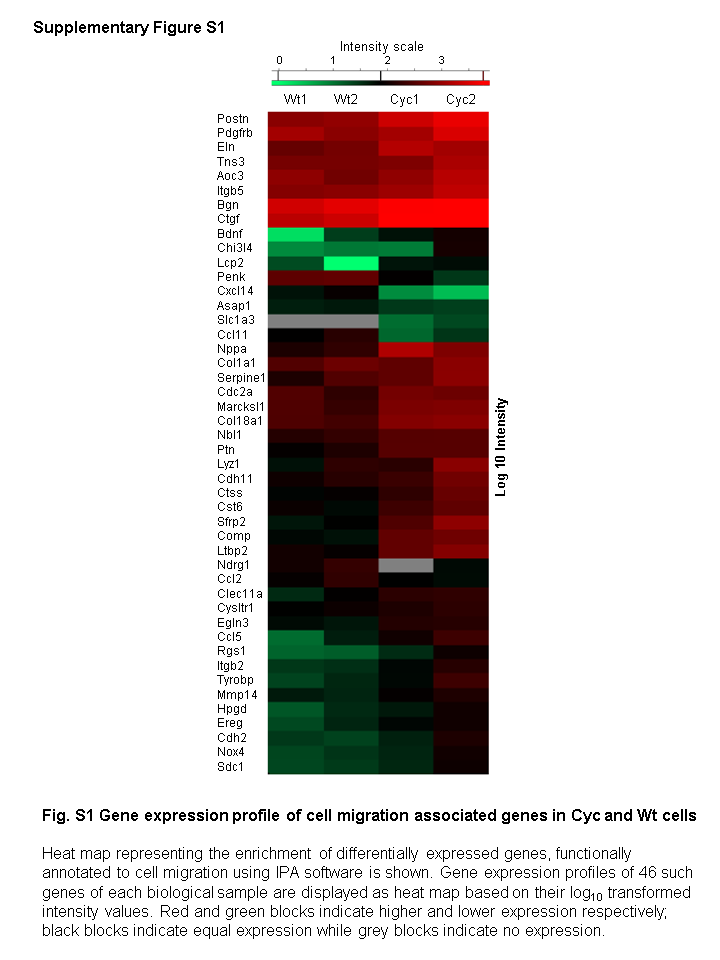

Supplement: S1 Fig — (TIF) [file pone.0120360.s001.TIF]

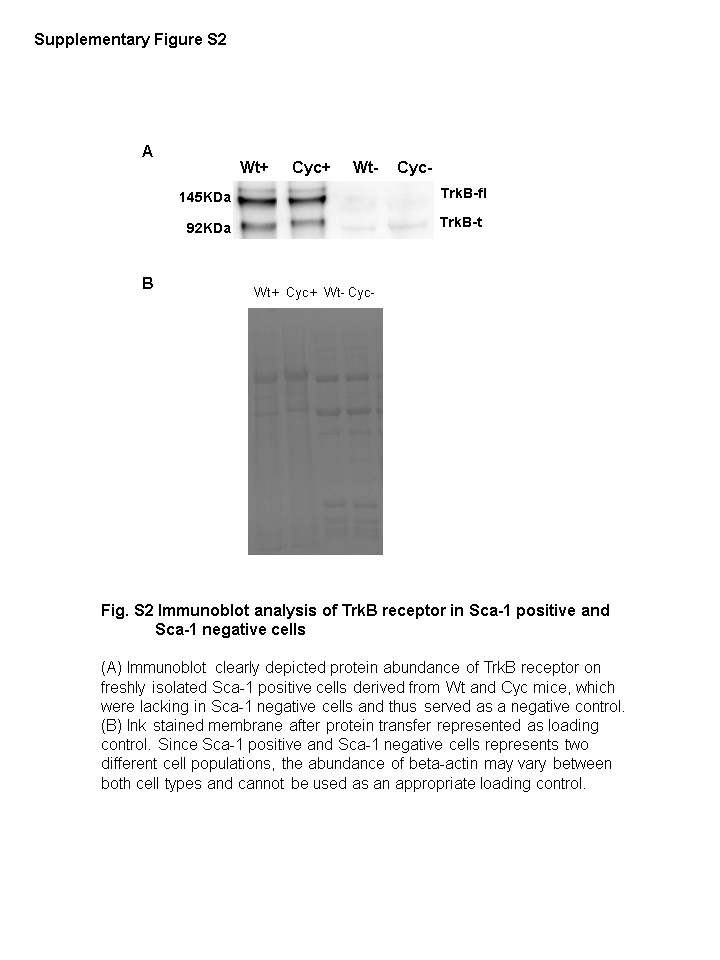

Supplement: S2 Fig — (TIF) [file pone.0120360.s002.TIF]

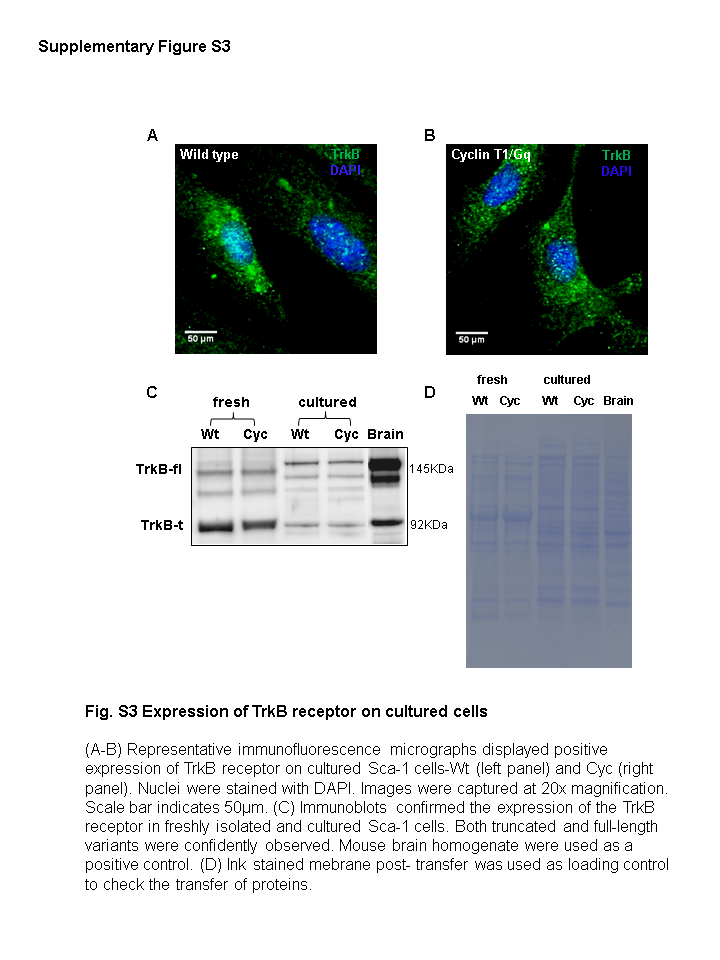

Supplement: S3 Fig — (TIF) [file pone.0120360.s003.TIF]

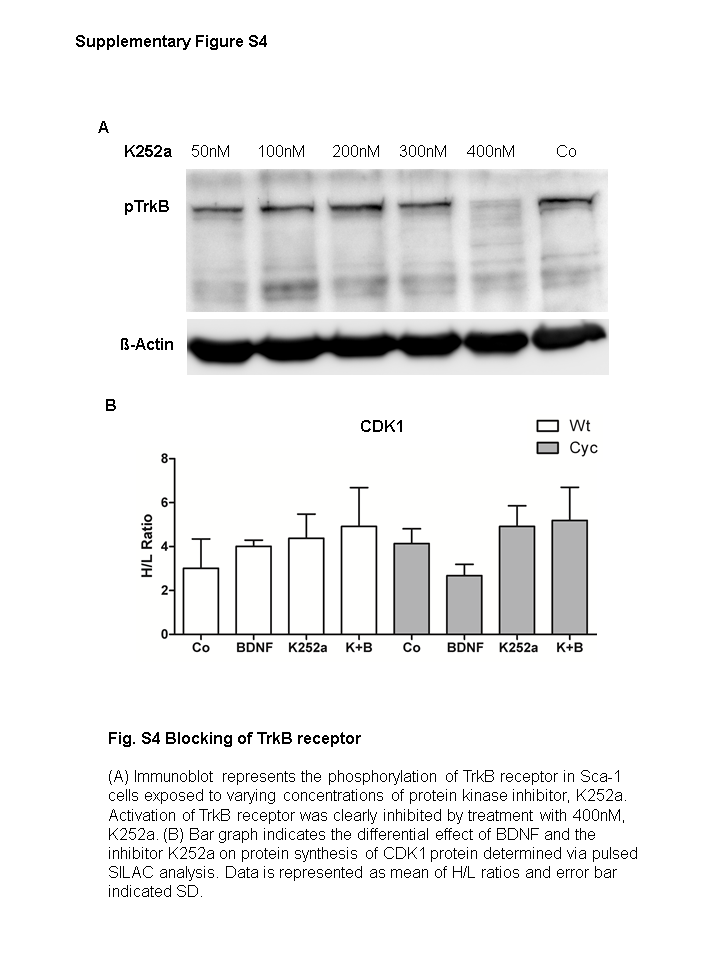

Supplement: S4 Fig — (TIF) [file pone.0120360.s004.TIF]

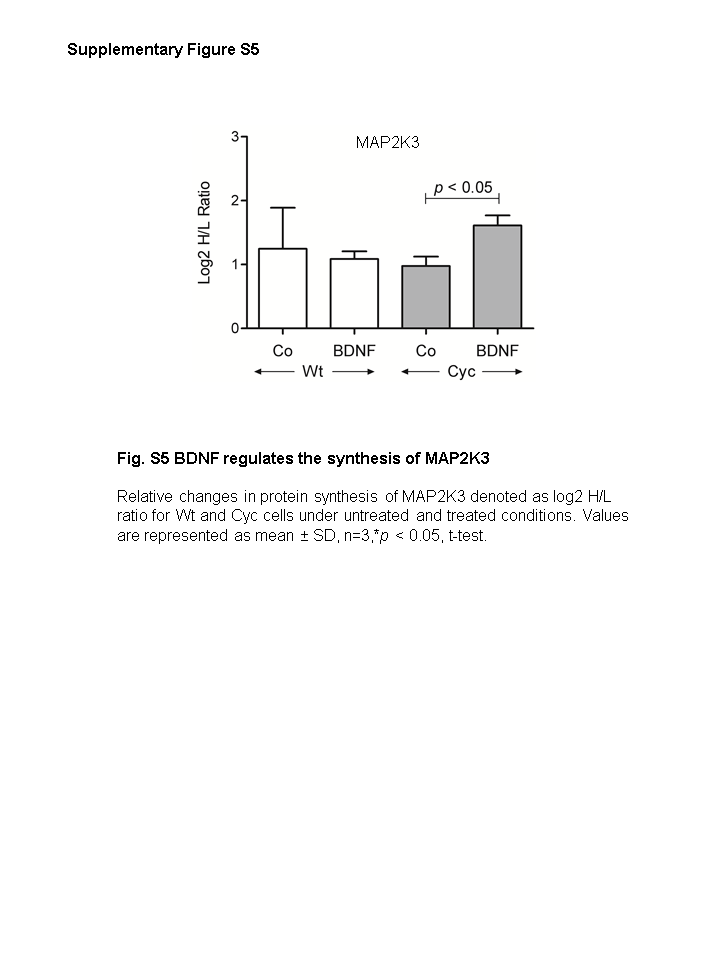

Supplement: S5 Fig — (TIF) [file pone.0120360.s005.TIF]
