# Supplementary material for: Brain Derived Neurotrophic Factor Contributes to the Cardiogenic Potential of Adult Resident Progenitor Cells in Failing Murine Heart
Source: PLoS One. 2015 Mar 23;10(3):e0120360. doi: 10.1371/journal.pone.0120360 (PMC4370398; doi:10.1371/journal.pone.0120360)
Supplement: S1 Table — (DOCX) [file pone.0120360.s006.docx]

**S1** **Table** **List of Taqman genes expression assays used in qRT PCR analysis**

| **Gene Symbol** | **Gene name** | **TaqMan Assays*** | **Microarray^+^** | **P-value** | **RT-PCR^+^** | **P-value** |
| --- | --- | --- | --- | --- | --- | --- |
| Actb | Beta-actin | Mm00607939_s1 |  |  |  |  |
| Bdnf | Brain derived neurotrophic factor | Mm01334042_m1 | 5.2 | <0.001 | 7.84 | 0.05 |
| Ccl19 | Chemokine (C-C motif) ligand 19 | Mm00839967_g1 | 2.96 | n.s. | 5.43 | 0.05 |
| Ccl9 | Chemokine (C-C motif) ligand 9 | Mm00441260_m1 | 2.9 | 0.011 | 47.18 | 0.05 |
| Crlf1 | Cytokine receptor-like factor 1 | Mm00517026_m1 | 18.37 | <0.001 | 16.87 | 0.05 |
| Cxcl13 | Chemokine (C-X-C motif) ligand 13 | Mm00444534_m1 | 2.32 | n.s. | 18.13 | 0.05 |
| Ptn | Pleiotrophin | Mm00436062_m1 | 2.9 | <0.001 | 3.6 | 0.05 |
| Sfrp2 | Secreted frizzled-related protein 2 | Mm01213947_m1 | 7.2 | <0.001 | 13.83 | 0.05 |
| Spp1 | Secreted phosphoprotein 1 | Mm00436767_m1 | 6.45 | n.s. | 39.49 | 0.05 |
| Wisp2 | WNT1 inducible signaling pathway protein 2 | Mm00497471_m1 | 11.0 | <0.001 | 21.86 | 0.05 |

* Taqman assay identity number supplied by Applied Biosystems

^+^ Fold change in expression of Cyc cells in comparison to Wt cells
