# Supplementary material for: Brain Derived Neurotrophic Factor Contributes to the Cardiogenic Potential of Adult Resident Progenitor Cells in Failing Murine Heart
Source: PLoS One. 2015 Mar 23;10(3):e0120360. doi: 10.1371/journal.pone.0120360 (PMC4370398; doi:10.1371/journal.pone.0120360)
Supplement: S2 Table — (DOCX) [file pone.0120360.s007.docx]

**S2 Table** **Gene expression of Cyclin T1 (Ccnt1), Gαq (Gnaq) and Sca-1 (Ly6a) in Sca-1 cells derived from transgenic and wildtype control mice**

No major difference in the expression of CyclinT1 (Ccnt1) or Gαq (Gnaq) was observed in Cyc cells compared to Wt as a result of overexpression. However, one out of nine probes for Gnaq showed significant changes. Since for all the other eight probe sets no difference in expression was detected, this result was assumed to be valid for the whole gene transcript**.**

| **Sequence Code** | **Primary Sequence Name** | **Intensity Profile data** | | | | **Fold change Cyc/Wt (Ratio)** | ***p*-value assuming presence of expression** | | | | ***p*-value assuming ratios (Cyc / Wt) Regulated expression** |
| --- | --- | --- | --- | --- | --- | --- | --- | --- | --- | --- | --- |
|  |  | **Cyc_BR1** | **Cyc_BR2** | **Wt_BR1** | **Wt_BR2** |  | **Cyc_BR1** | **Cyc_BR2** | **Wt_BR1** | **Wt_BR2** |  |
| 1450115_at | Gnaq | 723,6 | 492,3 | 22,7 | 4,1 | 19,06 | 0 | 0 | 0,09258 | 0,37827 | 1,71E-09 |
| 1458159_at | Gnaq | 75,5 | 352,6 | 115,4 | 365,3 | -1,03 | 8,94E-12 | 0 | 9,85E-20 | 0 |  |
| 1455729_at | Gnaq | 722,6 | 1410,0 | 1063,2 | 1344,4 | -1,16 | 0 | 0 | 0 | 0 |  |
| 1447593_x_at | Gnaq | 251,7 | 408,4 | 350,1 | 382,4 | -1,08 | 7,45E-34 | 4,48E-44 | 7,87E-41 | 4,31E-42 |  |
| 1446688_at | Gnaq | 27,0 | 74,3 | 68,6 | 59,0 | -1,07 | 0,02198 | 5,07E-07 | 4,43E-07 | 0,00002 |  |
| 1429559_at | Gnaq | 759,8 | 1580,0 | 1173,2 | 1689,9 | -1,24 | 0 | 0 | 0 | 0 |  |
| 1428938_at | Gnaq | 295,1 | 642,7 | 504,0 | 664,9 | -1,17 | 0 | 0 | 0 | 0 |  |
| 1428939_s_at | Gnaq | 696,1 | 1353,4 | 979,9 | 1398,3 | -1,08 | 0 | 0 | 0 | 0 |  |
| 1428940_at | Gnaq | 127,1 | 268,4 | 232,1 | 300,0 | -1,59 | 1,89E-14 | 1,84E-29 | 3,48E-28 | 8,13E-37 |  |
| 1419313_at | Ccnt1 | 39,2 | 56,3 | 48,4 | 70,1 | -1,31 | 0,01905 | 0,00159 | 0,00282 | 0,00001 |  |
| 1456477_at | Ccnt1 | 496,9 | 926,1 | 840,0 | 1086,3 | -1,36 | 0 | 0 | 0 | 0 |  |
| 1417185_at | Ly6a | 13368,3 | 11587,5 | 14050,6 | 10561,6 | -1,01 | 0 | 0 | 0 | 0 |  |

Fold change was calculated based on the normalized mean intensities of Cyc vs Wt cells. P-value was derived using the ratio measurement and its associated error (Rosetta Resolver error model).
