# Supplementary material for: Brain Derived Neurotrophic Factor Contributes to the Cardiogenic Potential of Adult Resident Progenitor Cells in Failing Murine Heart
Source: PLoS One. 2015 Mar 23;10(3):e0120360. doi: 10.1371/journal.pone.0120360 (PMC4370398; doi:10.1371/journal.pone.0120360)
Supplement: S3 Table — (DOCX) [file pone.0120360.s008.docx]

| **S3 Table** **Functional enrichment of biological processes in Cyc cells compared to Wt cells** | | | |
| --- | --- | --- | --- |
|  |  |  |  |
| **Molecular functions** | **z-score** | **P value** | **List of genes** |
| Angiogenesis | 1.9 | 3.84E-07 | AOC3,BDNF,CCL11,Ccl2,CCL5,CDH2,COL18A1,COL8A2,CTGF,CTSS,CXCL14,EGLN3,EREG,ITGB2,ITGB5,MB,MMP14,NOX4,NPPA,PDGFRB,PLVAP,PTN,SERPINE1,SFRP2 |
| Blood pressure | -1.5 | 1.23E-03 | ABCC9,AOC3,BDNF,BGN,COL1A2,EGLN3,ELN,NPPA,PTH1R,SERPINE1 |
| Ca^+2^ mobilization | 2.7 | 4.89E-03 | BDNF,CCL11,CCL5,CD79B,CYSLTR1,DIO2,ITGB2,LCP2,MS4A1 |
| Cardiovascular development | 2.49 | 4.72E-08 | AOC3,BDNF,BMPER,CCL11,Ccl2,CCL5,CDH2,COL18A1,COL1A1,COL1A2,COL8A2,CTGF,CTSS,CXCL14,EGLN3,ELN,EREG,ITGB2,MB,MMP14,NOX4,NPPA,PDGFRB,PLVAP,PTH1R,PTN,SERPINE1,SFRP2 |
| Cell activation | 2.24 | 1.17E-03 | BDNF,BMPER,Ccl2,CCL5,Ccl9,CD79B,CTGF,CTSS,DDIT4,GZMA,ITGB2,LCP2,MMP14,NDRG1,PDGFRB,PLTP,PTH1R,SBNO2,TYROBP |
| Cell aggregation | 2.57 | 5.00E-03 | BDNF,CDH11,CDH2,FZD2,GNAQ,ITGB2,LCP2,MMP14,PDGFRB |
| Cell migration | 2.35 | 7.58E-09 | AOC3,ASAP1,BDNF,BGN,CCL11,Ccl2,CCL5,CDH11,CDH2,CDK1,Chil3/Chil4,CLEC11A,COL18A1,COL1A1,COMP,CST6,CTGF,CTSS,CXCL14,CYSLTR1,EGLN3,ELN,EREG,HPGD,ITGB2,ITGB5,LCP2,LTBP2,Lyz1/Lyz2,MARCKSL1,MINOS1-NBL1/NBL1,MMP14,NDRG1,NOX4,NPPA,PDGFRB,PENK,POSTN,PTN,RGS1,SDC1,SERPINE1,SFRP2,SLC1A3,TNS3,TYROBP |
| Connective tissue development | 2.19 | 4.79E-04 | BGN,Ccl2,COL1A1,CTGF,MMP14,PDGFRB,POSTN,PTH1R,TYROBP |
| Dilation of heart | -2.0 | 1.46E-03 | BGN,MARCKSL1,NUPR1,SERPINE1 |
| Inflammation | -1.82 | 1.77E-03 | BGN,C1QA,CCL11,Ccl2,CCL5,CDH11,Chil3/Chil4,CLEC11A,COL1A1,COL1A2,COL4A4,CST6,CTSS,CYSLTR1,EREG,HPGD,ITGB2,ITGBL1,LCP2,MMP14,MS4A1,PDGFRB,POSTN,SERPINE1,TYROBP |
| Lipid metabolism | -2.18 | 3.75E-03 | BDNF,Ccl2,CTSS,CXCL14,DKK3,HPGD,NPPA,PLTP,PLVAP,PTH1R,SERPINE1,STEAP4 |
| Organismal death | -3.76 | 1.93E-04 | ABCC9,ADAM23,BACE2,BDNF,BGN,BMPER,C1QA,CCL11,Ccl2,CDH2,CDK1,COL12A1,COL1A1,COL5A2,CRLF1,CTGF,CXCL14,EGLN3,ELN,FABP3,GZMA,H19,HPGD,HUWE1,INHBB,IRF7,ITGB2,KIF1B,LCP2,LTBP2,Lyz1/Lyz2,MARCKSL1,MB,MMP14,MYL2,NDRG1,PDGFRB,PENK,PLVAP,POSTN,PTH1R,SERPINE1,SFRP2,SLC1A3,SLCO2A1,SPRED1,USP18 |
