# Supplementary material for: Brain Derived Neurotrophic Factor Contributes to the Cardiogenic Potential of Adult Resident Progenitor Cells in Failing Murine Heart
Source: PLoS One. 2015 Mar 23;10(3):e0120360. doi: 10.1371/journal.pone.0120360 (PMC4370398; doi:10.1371/journal.pone.0120360)
Supplement: S4 Table — (DOCX) [file pone.0120360.s009.docx]

**S4 Table** **List of differentially expressed genes in Cyc cells compared to Wt cells**

| **Gene Symbol** | **Annotation** | **Affymetrix ID** | **FC**  **(Cyc/Wt)** | ***p*-value** | **Types** |
| --- | --- | --- | --- | --- | --- |
|  | | | | | |
| **A. Genes encoding cytokines, growth factors and transcriptional regulators** | | | | | |
| ANKRD1 | Ankyrin repeat domain 1 (cardiac muscle) | 1420992_at | 4.4 | <0.001 | Transcription regulator |
| ATRX | Alpha thalassemia/mental retardation syndrome X-linked | 1420946_at | -2.1 | <0.001 | Transcription regulator |
| BDNF | Brain-derived neurotrophic factor | 1422168_a_at | 5.2 | <0.001 | Growth factor |
| CCL11 | Chemokine (C-C motif) ligand 11 | 1417789_at | -4.2 | 0.002 | Cytokine |
| CCL2 | Chemokine (C-C motif) ligand 2 | 1420380_at | -2.5 | 0.001 | Cytokine |
| CCL5 | Chemokine (C-C motif) ligand 5 | 1418126_at | 6.2 | <0.001 | Cytokine |
| CCL9 | Chemokine (C-C motif) ligand 9 | 1417936_at | 2.9 | 0.01 | Cytokine |
| CELF4 | CUGBP, Elav-like family member 4 | 1452240_at | 6.5 | <0.001 | Translation regulator |
| CLEC11A | C-type lectin domain family 11, member A | 1418796_at | 2.4 | 0.002 | Growth factor |
| CTGF | Connective tissue growth factor | 1416953_at | 3.2 | <0.001 | Growth factor |
| CXCL14 | Chemokine (C-X-C motif) ligand 14 | 1418457_at | -8.8 | <0.001 | Cytokine |
| CYTL1 | Cytokine-like 1 | 1456793_at | 2.4 | <0.001 | Cytokine |
| DKK3 | Dickkopf WNT signaling pathway inhibitor 3 | 1448669_at | 3.2 | <0.001 | Cytokine |
| EREG | Epiregulin | 1419431_at | 2.1 | <0.001 | Growth factor |
| FUBP1 | Far upstream element (FUSE) binding protein 1 | 1437544_at | -2.1 | 0.001 | Transcription regulator |
| HUWE1 | HECT, UBA and WWE domain containing 1, E3 ubiquitin protein ligase | 1444279_at | -2.3 | 0.002 | Transcription regulator |
| Ifi204 | Interferon activated gene 204 | 1452349_x_at | -3.3 | <0.001 | Transcription regulator |
| INHBB | Inhibin, beta B | 1426858_at | 2.3 | 0.001 | Growth factor |
| IRF7 | Interferon regulatory factor 7 | 1417244_a_at | 3.5 | <0.001 | Transcription regulator |
| NOTCH3 | Notch 3 | 1421965_s_at | 2.4 | <0.001 | Transcription regulator |
| NUPR1 | Nuclear protein, transcriptional regulator, 1 | 1419665_a_at | 2.3 | <0.001 | Transcription regulator |
| PTN | Pleiotrophin | 1448254_at | 2.9 | <0.001 | Growth factor |
| SBNO2 | Strawberry notch homolog 2 (Drosophila) | 1443721_x_at | 2.2 | <0.001 | Transcription regulator |
| WISP2 | WNT1 inducible signaling pathway protein 2 | 1419015_at | 11 | <0.001 | Growth factor |
|  |  |  |  |  |  |
| **B. Genes encoding receptors and surface antigens** | | |  |  |  |
| C1QTNF5 | C1q and tumor necrosis factor related protein 5 | 1424762_at | 2.4 | <0.001 | Transmembrane receptor |
| CD79B | CD79b molecule, immunoglobulin-associated beta | 1417640_at | 2.9 | 0.004 | Transmembrane receptor |
| CYSLTR1 | Cysteinyl leukotriene receptor 1 | 1449282_at | 2 | <0.001 | G-protein coupled receptor |
| FZD2 | Frizzled family receptor 2 | 1418534_at | 2.4 | <0.001 | G-protein coupled receptor |
| GPR160 | G protein-coupled receptor 160 | 1453072_at | -2.2 | 0.002 | G-protein coupled receptor |
| ITGB2 | Integrin, beta 2 (complement component 3 receptor 3 and 4 subunit) | 1450678_at | 2 | 0.002 | Transmembrane receptor |
| PTH1R | Parathyroid hormone 1 receptor | 1417092_at | 2 | <0.001 | G-protein coupled receptor |
| SFRP2 | Secreted frizzled-related protein 2 | 1448201_at | 7.2 | <0.001 | Transmembrane receptor |
| SSTR4 | Somatostatin receptor 4 | 1457440_at | -2.1 | <0.001 | G-protein coupled receptor |
| TYROBP | TYRO protein tyrosine kinase binding protein | 1450792_at | 4.7 | <0.001 | Transmembrane receptor |
|  |  |  |  |  |  |
| **C. Genes encoding kinases, peptidases, phosphatases and other enzymes** | | | |  |  |
| ADAM23 | ADAM metallopeptidase domain 23 | 1447946_at | -2.2 | <0.001 | Peptidase |
| ADAMTS2 | ADAM metallopeptidase with thrombospondin type 1 motif, 2 | 1435990_at | 2.4 | <0.001 | Peptidase |
| AOC3 | Amine oxidase, copper containing 3 | 1449396_at | 2 | <0.001 | Enzyme |
| ARL5B | ADP-ribosylation factor-like 5B | 1437884_at | -2.3 | 0.001 | Enzyme |
| BACE2 | Beta-site APP-cleaving enzyme 2 | 1437846_x_at | 2.3 | <0.001 | Peptidase |
| CDA | Cytidine deaminase | 1427357_at | 2.1 | 0.001 | Enzyme |
| CDK1 | Cyclin-dependent kinase 1 | 1448314_at | 2.2 | <0.001 | Kinase |
| CFI | Complement factor I | 1418724_at | 3.2 | <0.001 | Peptidase |
| Chil3/Chil4 | Chitinase-like 3 | 1425451_s_at | 5.5 | 0.002 | Enzyme |
| CILP | Cartilage intermediate layer protein, nucleotide pyrophosphohydrolase | 1457296_at | 2.5 | <0.001 | Phosphatase |
| CTSS | Cathepsin S | 1448591_at | 4.4 | <0.001 | Peptidase |
| DIO2 | Deiodinase, iodothyronine, type II | 1418937_at | 2.6 | <0.001 | Enzyme |
| EGLN3 | Egl-9 family hypoxia-inducible factor 3 | 1418649_at | 2.5 | <0.001 | Enzyme |
| ELOVL7* | ELOVL fatty acid elongase 7 | 1424098_at | -2.2 | 0.03 | Enzyme |
| ENPP1 | Ectonucleotide pyrophosphatase/phosphodiesterase 1 | 1440339_at | 2.5 | 0.001 | Enzyme |
| ENPP6 | Ectonucleotide pyrophosphatase/phosphodiesterase 6 | 1438785_at | 3 | <0.001 | Enzyme |
| EPHX3 | Epoxide hydrolase 3 | 1430724_at | 2.2 | <0.001 | Enzyme |
| FGL2 | Fibrinogen-like 2 | 1421855_at | 3.1 | <0.001 | Peptidase |
| GNG8 | Guanine nucleotide binding protein (G protein), gamma 8 | 1457755_at | 2.6 | <0.001 | Enzyme |
| GZMA | Granzyme A (granzyme 1, cytotoxic T-lymphocyte-associated serine esterase 3) | 1417898_a_at | 2.5 | <0.001 | Peptidase |
| HPGD | Hydroxyprostaglandin dehydrogenase 15-(NAD) | 1419905_s_at | 2.5 | 0.001 | Enzyme |
| INMT | Indolethylamine N-methyltransferase | 1418697_at | -2.3 | 0.001 | Enzyme |
| LOXL3 | Lysyl oxidase-like 3 | 1418269_at | 2.1 | <0.001 | Enzyme |
| LTF | Lactotransferrin | 1450009_at | 10.5 | 0.002 | Peptidase |
| LYZ1/LYZ2 | Lysozyme 2 | 1439426_x_at | 4 | 0.001 | Enzyme |
| MASP1 | Mannan-binding lectin serine peptidase 1 | 1438602_s_at | 2.8 | <0.001 | Peptidase |
| MMP14 | Matrix metallopeptidase 14 | 1440920_at | 2.1 | 0.002 | Peptidase |
| MMP23B | Matrix metallopeptidase 23B | 1417281_a_at | 3.9 | <0.001 | Peptidase |
| MOXD1 | Monooxygenase, DBH-like 1 | 1422643_at | 2.4 | <0.001 | Enzyme |
| NDRG1 | N-myc downstream regulated 1 | 1450977_s_at | -2.4 | 0.007 | Kinase |
| NOX4 | NADPH oxidase 4 | 1419161_a_at | 2.6 | 0.008 | Enzyme |
| OAS1 | 2'-5'-oligoadenylate synthetase 1, 40/46kDa | 1424775_at | 2.4 | <0.001 | Enzyme |
| OASL | 2'-5'-oligoadenylate synthetase-like | 1424339_at | 2.4 | <0.001 | Enzyme |
| OASL2 | 2'-5' oligoadenylate synthetase-like 2 | 1453196_a_at | 2.3 | <0.001 | Enzyme |
| PALD1 | Phosphatase domain containing, paladin 1 | 1421811_at | 2.1 | <0.001 | Phosphatase |
| PDGFRB | Platelet-derived growth factor receptor, beta polypeptide | 1436970_a_at | 2 | 0.001 | Kinase |
| PDGFRL | Platelet-derived growth factor receptor-like | 1428896_at | 2.3 | <0.001 | Kinase |
| PDZRN3 | PDZ domain containing ring finger 3 | 1416846_a_at | 2.7 | <0.001 | Enzyme |
| PLTP | Phospholipid transfer protein | 1417963_at | -2.2 | <0.001 | Enzyme |
| RASL11B | RAS-like, family 11, member B | 1423854_a_at | 3 | <0.001 | Enzyme |
| SDC1 | Syndecan 1 | 1415944_at | 3.3 | <0.001 | Enzyme |
| STEAP4 | STEAP family member 4 | 1460197_a_at | 2 | 0.001 | Enzyme |
| TNS3 | Tensin 3 | 1455333_at | 2.1 | <0.001 | Phosphatase |
| USP18 | Ubiquitin specific peptidase 18 | 1418191_at | 2.8 | <0.001 | Peptidase |
|  |  |  |  |  |  |
| **D. Genes encoding ion channels and transporters** | | |  |  |  |
| ABCC9 | ATP-binding cassette, sub-family C (CFTR/MRP), member 9 | 1420408_a_at | 2.1 | <0.001 | Ion channel |
| FABP3 | Fatty acid binding protein 3, muscle and heart (mammary-derived growth inhibitor) | 1416023_at | -2.6 | <0.001 | Transporter |
| KCNE3 | Potassium voltage-gated channel, Isk-related family, member 3 | 1418499_a_at | 3.2 | <0.001 | Ion channel |
| KIF1B | Kinesin family member 1B | 1423995_at | -2.5 | 0.001 | Transporter |
| MB | Myoglobin | 1451203_at | -2.4 | <0.001 | Transporter |
| NXNL2* | Nucleoredoxin-like 2 | 1429133_at | 2.2 | 0.021 | Transporter |
| RBP1 | Retinol binding protein 1, cellular | 1448754_at | 3.2 | <0.001 | Transporter |
| SEC14L3 | SEC14-like 3 (S. cerevisiae) | 1436787_x_at | 10.3 | <0.001 | Transporter |
| SLC1A3 | Solute carrier family 1 (glial high affinity glutamate transporter), member 3 | 1452031_at | 6.9 | 0.001 | Transporter |
| SLC40A1 | Solute carrier family 40 (iron-regulated transporter), member 1 | 1417061_at | 2.7 | 0.002 | Transporter |
| SLCO2A1 | Solute carrier organic anion transporter family, member 2A1 | 1420913_at | 2 | <0.001 | Transporter |
|  |  |  |  |  |  |
| **E. Other differentially expressed genes** | |  |  |  |  |
| 1110002E22Rik | RIKEN cDNA 1110002E22 gene | 1447870_x_at | 4.8 | 0.011 | Other |
| 2010300C02Rik | RIKEN cDNA 2010300C02 gene | 1452861_at | 2.6 | 0.009 | Other |
| 2010309G21Rik | RIKEN cDNA 2010309G21 gene | 1428719_at | 5.8 | <0.001 | Other |
| AARD* | Alanine and arginine rich domain containing protein | 1434528_at | 3.5 | 0.033 | Other |
| ADAMTSL2 | ADAMTS-like 2 | 1429214_at | 4.4 | <0.001 | Other |
| ANGPTL7 | Angiopoietin-like 7 | 1451478_at | 4.4 | 0.002 | Other |
| ANKRD11 | Ankyrin repeat domain 11 | 1456110_at | -2.1 | 0.001 | Other |
| APCDD1 | Adenomatosis polyposis coli down-regulated 1 | 1454822_x_at | 2.1 | <0.001 | Other |
| ASAP1* | ArfGAP with SH3 domain, ankyrin repeat and PH domain 1 | 1425573_a_at | -2.0 | 0.015 | Other |
| AU015791 | Expressed sequence AU015791 | 1442012_at | 2.7 | <0.001 | Other |
| AU040972 | Expressed sequence AU040972 | 1443946_s_at | 10.1 | <0.001 | Other |
| B230217C12Rik* | RIKEN cDNA B230217C12 gene | 1428568_at | 2.2 | 0.023 | Other |
| BC023969 | cDNA sequence BC023969 | 1445226_at | -2.1 | 0.001 | Other |
| BEND7* | BEN domain containing 7 | 1439186_at | -5.9 | 0.021 | Other |
| BGN | Biglycan | 1448323_a_at | 2.2 | <0.001 | Other |
| BMPER | BMP binding endothelial regulator | 1429273_at | -2.3 | <0.001 | Other |
| BST2 | Bone marrow stromal cell antigen 2 | 1424921_at | 2.4 | 0.001 | Other |
| C11orf96 | Chromosome 11 open reading frame 96 | 1455271_at | 2.3 | <0.001 | Other |
| C15orf48 | Chromosome 15 open reading frame 48 | 1434046_at | 3.2 | 0.004 | Other |
| C1QA | Complement component 1, q subcomponent, A chain | 1417381_at | 16.4 | <0.001 | Other |
| C1QB | Complement component 1, q subcomponent, B chain | 1437726_x_at | 7.2 | 0.002 | Other |
| C2orf40 | Chromosome 2 open reading frame 40 | 1460049_s_at | 16.0 | <0.001 | Other |
| CCSER2 | Coiled-coil serine-rich protein 2 | 1453240_a_at | -2.0 | <0.001 | Other |
| CD52 | CD52 antigen | 1460218_at | 2.7 | <0.001 | Other |
| CDH11 | Cadherin 11, type 2, OB-cadherin (osteoblast) | 1450757_at | 2.6 | <0.001 | Other |
| CDH2 | Cadherin 2, type 1, N-cadherin (neuronal) | 1418815_at | 2.8 | <0.001 | Other |
| CEP135* | Centrosomal protein 135kDa | 1444201_at | -2.5 | 0.050 | Other |
| CGREF1* | Cell growth regulator with EF-hand domain 1 | 1424529_s_at | 2.7 | 0.033 | Other |
| COL12A1* | Collagen, type XII, alpha 1 | 1434411_at | 3.4 | 0.013 | Other |
| COL18A1 | Collagen, type XVIII, alpha 1 | 1418237_s_at | 2.9 | <0.001 | Other |
| COL1A1 | Collagen, type I, alpha 1 | 1423669_at | 3.9 | <0.001 | Other |
| COL1A2 | Collagen, type I, alpha 2 | 1423110_at | 2.6 | <0.001 | Other |
| COL4A4 | Collagen, type IV, alpha 4 | 1445328_at | 2.9 | <0.001 | Other |
| COL5A2 | Collagen, type V, alpha 2 | 1450625_at | 2.4 | <0.001 | Other |
| COL6A2 | Collagen, type VI, alpha 2 | 1452250_a_at | 2.3 | <0.001 | Other |
| COL8A2 | Collagen, type VIII, alpha 2 | 1434667_at | 5.0 | <0.001 | Other |
| COLEC11 | Collectin sub-family member 11 | 1458345_s_at | 3.4 | <0.001 | Other |
| COMP | Cartilage oligomeric matrix protein | 1419527_at | 9.1 | <0.001 | Other |
| CRLF1 | Cytokine receptor-like factor 1 | 1418476_at | 18.4 | <0.001 | Other |
| CST6 | Cystatin E/M | 1427910_at | 4.3 | <0.001 | Other |
| DDIT4 | DNA-damage-inducible transcript 4 | 1428306_at | 2.5 | <0.001 | Other |
| DDX3Y | DEAD (Asp-Glu-Ala-Asp) box polypeptide 3, Y-linked | 1426439_at | -3.0 | 0.001 | Other |
| DYNLRB2 | Dynein, light chain, roadblock-type 2 | 1428987_at | 9.0 | 0.007 | Other |
| EAR3* | Eosinophil-associated, ribonuclease A family, member 3 | 1422412_x_at | 4.7 | 0.025 | Other |
| ELN | Elastin | 1420855_at | 2.8 | <0.001 | Other |
| ENHO | Energy homeostasis associated | 1428739_at | 6.1 | <0.001 | Other |
| EXOC3L4 | Exocyst complex component 3-like 4 | 1428420_a_at | 3.6 | <0.001 | Other |
| FAM167B | Family with sequence similarity 167, member B | 1455587_at | 7.8 | <0.001 | Other |
| FAM26E | Family with sequence similarity 26, member E | 1424680_at | 2.0 | <0.001 | Other |
| FAM84A | Family with sequence similarity 84, member A | 1425452_s_at | 7.6 | 0.001 | Other |
| FIBIN | Fin bud initiation factor homolog (zebrafish) | 1419376_at | 2.1 | <0.001 | Other |
| FMOD | Fibromodulin | 1456084_x_at | 10.0 | <0.001 | Other |
| FRZB | Frizzled-related protein | 1416658_at | 3.9 | <0.001 | Other |
| GLYCAM1 | Glycosylation dependent cell adhesion molecule 1 | 1424825_a_at | 41.2 | 0.006 | Other |
| H19 | H19, imprinted maternally expressed transcript (non-protein coding) | 1448194_a_at | 5.9 | <0.001 | Other |
| Hist1h2ab | Histone cluster 1, H2ab | 1438009_at | 2.0 | <0.001 | Other |
| IFI27L2 | Interferon, alpha-inducible protein 27-like 2 | 1426278_at | 3.5 | <0.001 | Other |
| IFI44 | Interferon-induced protein 44 | 1423555_a_at | 2.9 | <0.001 | Other |
| IFIT1B | Interferon-induced protein with tetratricopeptide repeats 1B | 1450783_at | 3.0 | <0.001 | Other |
| IFIT3 | Interferon-induced protein with tetratricopeptide repeats 3 | 1449025_at | 4.0 | <0.001 | Other |
| ITGB5 | Integrin, beta 5 | 1417533_a_at | 2.4 | <0.001 | Other |
| ITGBL1 | Integrin, beta-like 1 (with EGF-like repeat domains) | 1425039_at | 4.7 | <0.001 | Other |
| ITIH2 | Inter-alpha-trypsin inhibitor heavy chain 2 | 1417618_at | 3.8 | <0.001 | Other |
| ITIH4 | Inter-alpha-trypsin inhibitor heavy chain family, member 4 | 1431808_a_at | 5.1 | 0.002 | Other |
| ITIH5 | Inter-alpha-trypsin inhibitor heavy chain family, member 5 | 1436755_at | 2.3 | <0.001 | Other |
| ITLN1 | Intelectin 1 (galactofuranose binding) | 1418165_at | 12.2 | 0.007 | Other |
| KIAA1033 | KIAA1033 | 1427319_at | -2.1 | <0.001 | Other |
| LANCL3 | LanC lantibiotic synthetase component C-like 3 (bacterial) | 1437268_at | -2.3 | 0.004 | Other |
| LCP2 | Lymphocyte cytosolic protein 2 (SH2 domain containing leukocyte protein of 76kDa) | 1418641_at | 2.3 | 0.002 | Other |
| LDLRAD4* | Low density lipoprotein receptor class A domain containing 4 | 1452343_at | 2.1 | 0.022 | Other |
| LTBP2 | Latent transforming growth factor beta binding protein 2 | 1418061_at | 3.8 | <0.001 | Other |
| MARCKSL1 | MARCKS-like 1 | 1437226_x_at | 2.1 | <0.001 | Other |
| MFAP4 | Microfibrillar-associated protein 4 | 1424010_at | 4.7 | <0.001 | Other |
| MFAP5 | Microfibrillar associated protein 5 | 1449082_at | 2.2 | <0.001 | Other |
| MINOS1-NBL1/NBL1 | Neuroblastoma 1, DAN family BMP antagonist | 1448428_at | 2.3 | <0.001 | Other |
| MPEG1 | Macrophage expressed 1 | 1427076_at | 12.8 | <0.001 | Other |
| MS4A1 | Membrane-spanning 4-domains, subfamily A, member 1 | 1423226_at | 3.4 | 0.002 | Other |
| MYL2 | Myosin, light chain 2, regulatory, cardiac, slow | 1448394_at | -2.3 | <0.001 | Other |
| MYL3 | Myosin, light chain 3, alkali; ventricular, skeletal, slow | 1427768_s_at | -2.5 | <0.001 | Other |
| NKD2 | Naked cuticle homolog 2 (Drosophila) | 1434275_at | 3.1 | <0.001 | Other |
| NPPA | Natriuretic peptide A | 1456062_at | 6.2 | <0.001 | Other |
| PCDH9 | Protocadherin 9 | 1429861_at | 2.1 | 0.001 | Other |
| PCDHB9 | Protocadherin beta 9 | 1422640_at | 2.1 | 0.001 | Other |
| PDLIM3 | PDZ and LIM domain 3 | 1449178_at | 2.2 | 0.001 | Other |
| PENK | Proenkephalin | 1427038_at | -5.0 | <0.001 | Other |
| PKIB* | Protein kinase (cAMP-dependent, catalytic) inhibitor beta | 1421137_a_at | 3.7 | 0.033 | Other |
| PKIG | Protein kinase (cAMP-dependent, catalytic) inhibitor gamma | 1423945_a_at | 2.0 | <0.001 | Other |
| PLET1 | Placenta expressed transcript 1 | 1423933_a_at | 8.0 | 0.009 | Other |
| PLVAP | Plasmalemma vesicle associated protein | 1418090_at | 2.3 | <0.001 | Other |
| POSTN | Periostin, osteoblast specific factor | 1423606_at | 3.6 | <0.001 | Other |
| PRRC2C | Proline-rich coiled-coil 2C | 1429432_at | -2.0 | 0.001 | Other |
| RGS1 | Regulator of G-protein signaling 1 | 1417601_at | 2.7 | 0.004 | Other |
| RTP4 | Receptor (chemosensory) transporter protein 4 | 1418580_at | 2.1 | <0.001 | Other |
| SCGB3A2 | Secretoglobin, family 3A, member 2 | 1425218_a_at | 12.6 | <0.001 | Other |
| SERPINE1 | Serpin peptidase inhibitor, clade E (nexin, plasminogen activator inhibitor type 1), member 1 | 1419149_at | 4.2 | <0.001 | Other |
| SPRED1 | Sprouty-related, EVH1 domain containing 1 | 1423162_s_at | -2.1 | <0.001 | Other |
| STC2 | Stanniocalcin 2 | 1419503_at | 5.0 | <0.001 | Other |
| SVEP1 | Sushi, von Willebrand factor type A, EGF and pentraxin domain containing 1 | 1419182_at | 4.6 | <0.001 | Other |
| SYNPO | Synaptopodin | 1434089_at | 2.3 | <0.001 | Other |
| SYNPO2L | Synaptopodin 2-like | 1447657_s_at | 3.0 | <0.001 | Other |
| TMEM150C | Transmembrane protein 150C | 1436656_at | 3.4 | <0.001 | Other |
| TMEM182 | Transmembrane protein 182 | 1428899_at | -2.2 | <0.001 | Other |
| TMEM252 | Transmembrane protein 252 | 1437595_at | 3.9 | <0.001 | Other |
| TRBV1 | T cell receptor beta, variable 1 | 1452205_x_at | -2.6 | 0.004 | Other |
| UTY | Ubiquitously transcribed tetratricopeptide repeat gene, Y chromosome | 1457582_at | -3.9 | 0.005 | Other |
| VGLL3 | Vestigial like 3 (Drosophila) | 1453593_at | 2.0 | <0.001 | Other |
| ZBTB7C | Zinc finger and BTB domain containing 7C | 1436365_at | 4.1 | 0.001 | Other |

Differentially expressed genes were assumed with fold change (FC) difference of atleast 2. FC was calculated based on the normalized mean intensities of cyclin cells over wildtype cells. The set of genes listed above display a *p* value < 0.01(Rosetta Resolver error model) which is derived from the ratio measurement and its associated error,**p* < 0.03. Gene symbol and annotation were taken from Resolver database (2011).
