# Supplementary material for: Brain Derived Neurotrophic Factor Contributes to the Cardiogenic Potential of Adult Resident Progenitor Cells in Failing Murine Heart
Source: PLoS One. 2015 Mar 23;10(3):e0120360. doi: 10.1371/journal.pone.0120360 (PMC4370398; doi:10.1371/journal.pone.0120360)
Supplement: S5 Table — Data represents average of log2 transformed H/L ratios. (DOCX) [file pone.0120360.s010.docx]

**S5 Table** **K-means clustering of BDNF- mediated regulated proteins in Cyc and Wt cells. Data represents average of log2 transformed H/L ratios**

| **Accession** | **Annotation** | **Protein name** | **Cyc BDNF** | **Cyc Co** | **Log2 Ratio (BDNF/Co)** | **Ratio (BDNF/Co)** | **P value** |
| --- | --- | --- | --- | --- | --- | --- | --- |
| **Cluster 1** | **BDNF induced** |  |  |  |  |  |  |
| Q9JKV1 | Proteasomal ubiquitin receptor ADRM1 | ADRM1 | 1.15 | 0.73 | 0.43 | 1.35 | 0.048 |
| Q61545 | RNA-binding protein EWS | EWS | 0.36 | -0.02 | 0.38 | 1.30 | 0.038 |
| P23591 | GDP-L-fucose synthase | FCL | 0.20 | -0.12 | 0.32 | 1.25 | 0.004 |
| Q9Z2Z9 | Glucosamine-fructose-6-phosphate aminotransferase [isomerizing] 2 | GFPT2 | 0.69 | 0.36 | 0.33 | 1.26 | 0.038 |
| P51859 | Hepatoma-derived growth factor | HDGF | 0.28 | -0.46 | 0.75 | 1.68 | 0.006 |
| Q05D44 | Eukaryotic translation initiation factor 5B | IF2P | 0.69 | 0.24 | 0.46 | 1.37 | 0.037 |
| Q91VC3 | Eukaryotic initiation factor 4A-III | IF4A3 | 1.34 | 0.79 | 0.55 | 1.46 | 0.033 |
| Q62448 | Eukaryotic translation initiation factor 4 gamma 2 | IF4G2 | 0.68 | 0.31 | 0.36 | 1.29 | 0.011 |
| O09110 | Dual specificity mitogen-activated protein kinase kinase 3 | MP2K3 | 1.60 | 0.98 | 0.62 | 1.53 | 0.016 |
| Q8VCM8 | Nicalin | NCLN | 0.46 | -0.40 | 0.86 | 1.82 | 0.032 |
| Q9Z0W3 | Nuclear pore complex protein Nup160 | NU160 | 0.21 | -0.33 | 0.55 | 1.46 | 0.038 |
| Q9WVE8 | Protein kinase C and casein kinase substrate in neurons protein 2 | PACN2 | 0.68 | 0.23 | 0.45 | 1.37 | 0.010 |
| P83870 | PHD finger-like domain-containing protein 5A | PHF5A | 0.89 | 0.37 | 0.51 | 1.43 | 0.023 |
| P97372 | Proteasome activator complex subunit 2 | PSME2 | 0.74 | 0.26 | 0.48 | 1.40 | 0.022 |
| Q8CI94 | Glycogen phosphorylase, brain form | PYGB | 1.93 | 0.23 | 1.71 | 3.26 | 0.011 |
| Q62241 | U1 small nuclear ribonucleoprotein C | RU1C | 1.57 | 1.02 | 0.54 | 1.46 | 0.014 |
| Q9ERD7 | Tubulin beta-3 chain | TBB3 | 0.94 | 0.61 | 0.32 | 1.25 | 0.006 |
|  |  |  |  |  |  |  |  |
|  |  |  |  |  |  |  |  |
| **Accession** | **Annotation** | **Protein name** | **Cyc BDNF** | **Cyc Co** | **Log2 Ratio (BDNF/Co)** | **Ratio (BDNF/Co)** | **P value** |
| Q8BWT1 | 3-ketoacyl-CoA thiolase, mitochondrial | THIM | -0.19 | -0.79 | 0.60 | 1.51 | 0.024 |
| P12032 | Metalloproteinase inhibitor 1 | TIMP1 | 1.87 | 1.09 | 0.78 | 1.72 | 0.011 |
| O55013 | Trafficking protein particle complex subunit 3 | TPPC3 | 1.12 | 0.08 | 1.04 | 2.06 | 0.026 |
| Q9WV55 | Vesicle-associated membrane protein-associated protein A | VAPA | 0.30 | -0.12 | 0.43 | 1.34 | 0.043 |
| **Cluster 2** | **BDNF repressed** |  |  |  |  |  |  |
| Q923D2 | Flavin reductase | BLVRB | -0.14 | 0.17 | -0.31 | 0.81 | 0.034 |
| P15379 | CD44 antigen | CD44 | 0.78 | 1.35 | -0.57 | 0.67 | 0.001 |
| Q8R349 | Cell division cycle protein 16 homolog | CDC16 | 1.23 | 1.73 | -0.50 | 0.71 | 0.032 |
| P11440 | Cyclin-dependent kinase 1 | CDK1 | 1.46 | 2.19 | -0.73 | 0.60 | 0.039 |
| Q6PEE2 | CBP80/20-dependent translation initiation factor | CTIF | 0.78 | 1.18 | -0.40 | 0.76 | 0.007 |
| Q91YW3 | DnaJ homolog subfamily C member 3 | DNJC3 | 0.13 | 0.48 | -0.35 | 0.79 | 0.037 |
| P21550 | Beta-enolase | ENOB | 0.19 | 0.56 | -0.37 | 0.77 | 0.016 |
| P21278 | Guanine nucleotide-binding protein subunit alpha-11 | GNA11 | 0.54 | 0.87 | -0.33 | 0.79 | 0.024 |
| Q99LJ6 | Glutathione peroxidase 7 | GPX7 | -0.85 | -0.35 | -0.49 | 0.71 | 0.025 |
| Q04447 | Creatine kinase B-type | KCRB | 0.18 | 0.50 | -0.32 | 0.80 | 0.010 |
| Q501J7 | Phosphatase and actin regulator 4 | PHAR4 | 0.23 | 1.54 | -1.31 | 0.40 | 0.029 |
| P54822 | Adenylosuccinate lyase | PUR8 | -0.12 | 0.19 | -0.32 | 0.80 | 0.045 |
| Q91YJ2 | Sorting nexin-4 | SNX4 | 0.73 | 1.29 | -0.56 | 0.68 | 0.009 |
| Q5I2A0 | Serine protease inhibitor A3G | SPA3G | 2.99 | 3.45 | -0.46 | 0.73 | 0.016 |
| Q99MR6 | Serrate RNA effector molecule homolog | SRRT | 0.46 | 1.26 | -0.80 | 0.58 | 0.020 |
| Q99J36 | THUMP domain-containing protein 1 | THUM1 | 0.50 | 1.18 | -0.68 | 0.62 | 0.014 |
| P70398 | Probable ubiquitin carboxyl-terminal hydrolase FAF-X | USP9X | 0.49 | 1.03 | -0.53 | 0.69 | 0.014 |
|  |  |  |  |  |  |  |  |
| **Accession** | **Annotation** | **Protein name** | **Wt BDNF** | **Wt Co** | **Log2 Ratio (BDNF/Co)** | **Ratio (BDNF/Co)** | **P value** |
| **Cluster1** | **BDNF induced** |  |  |  |  |  |  |
|  |  |  |  |  |  |  |  |
| O35841 | Apoptosis inhibitor 5 | API5 | 1.40 | 0.60 | 0.80 | 1.74 | 0.009 |
| Q8BJF9 | Charged multivesicular body protein 2b | CHM2B | 0.28 | -0.08 | 0.35 | 1.28 | 0.039 |
| Q8K1M6 | Dynamin-1-like protein | DNM1L | 0.96 | 0.63 | 0.33 | 1.25 | 0.032 |
| Q9CY57 | Friend of PRMT1 protein | FOP | 1.22 | 0.66 | 0.56 | 1.47 | 0.014 |
| P14901 | Heme oxygenase 1 | HMOX1 | 3.48 | 2.70 | 0.78 | 1.72 | 0.014 |
| Q8BUK6 | Protein Hook homolog 3 | HOOK3 | 0.86 | 0.51 | 0.35 | 1.27 | 0.002 |
| P02468 | Laminin subunit gamma-1 | LAMC1 | 3.51 | 2.40 | 1.11 | 2.16 | 0.044 |
| Q8R2Y8 | Peptidyl-tRNA hydrolase 2, mitochondrial | PTH2 | 0.79 | 0.41 | 0.38 | 1.30 | 0.005 |
| Q8VDQ8 | NAD-dependent protein deacetylase sirtuin-2 | SIR2 | 0.88 | -0.35 | 1.24 | 2.36 | 0.013 |
| Q99MR6 | Serrate RNA effector molecule homolog | SRRT | 1.16 | 0.67 | 0.49 | 1.41 | 0.046 |
| Q8C1E7 | Transmembrane protein 120A | T120A | -1.00 | -1.86 | 0.86 | 1.82 | 0.022 |
| **Cluster 2** | **BDNF repressed** |  |  |  |  |  |  |
|  |  |  |  |  |  |  |  |
| Q9EPJ9 | ADP-ribosylation factor GTPase-activating protein 1 | ARFG1 | 0.83 | 1.54 | -0.71 | 0.61 | 0.027 |
| O70503 | Estradiol 17-beta-dehydrogenase 12 | DHB12 | -0.16 | 0.21 | -0.37 | 0.78 | 0.012 |
| Q8BTZ7 | Mannose-1-phosphate guanyltransferase beta | GMPPB | 0.18 | 0.64 | -0.46 | 0.72 | 0.036 |
| O35166 | Golgi SNAP receptor complex member 2 | GOSR2 | 0.80 | 1.61 | -0.81 | 0.57 | 0.046 |
| P19788 | Matrix Gla protein | MGP | -0.42 | 0.61 | -1.03 | 0.49 | 0.012 |
| P03921 | NADH-ubiquinone oxidoreductase chain 5 | NU5M | 0.12 | 0.61 | -0.49 | 0.71 | 0.048 |
| Q9WU28 | Prefoldin subunit 5 | PFD5 | 0.29 | 0.65 | -0.36 | 0.78 | 0.034 |
| **Accession** | **Annotation** | **Protein name** | **Wt BDNF** | **Wt Co** | **Log2 Ratio (BDNF/Co)** | **Ratio (BDNF/Co)** | **P value** |
|  |  |  |  |  |  |  |  |
| B2RXS4 | Plexin-B2 | PLXB2 | 1.43 | 2.12 | -0.68 | 0.62 | 0.044 |
| P97372 | Proteasome activator complex subunit 2 | PSME2 | 0.32 | 0.80 | -0.48 | 0.72 | 0.009 |
| Q9DCF9 | Translocon-associated protein subunit gamma | SSRG | 2.01 | 2.55 | -0.53 | 0.69 | 0.031 |
| Q8VCK3 | Tubulin gamma-2 chain | TBG2 | -0.58 | 0.43 | -1.01 | 0.50 | 0.009 |
